# Supplementary material for: Effects of discontinuation of levothyroxine treatment in older adults: protocol for a self-controlled trial
Source: BMJ Open. 2023 Apr 25;13(4):e070741. doi: 10.1136/bmjopen-2022-070741 (PMC10151847; doi:10.1136/bmjopen-2022-070741)
Supplement: Supplementary data [file bmjopen-2022-070741supp001.pdf]

Appendix 1

Subject information for participation in medical research

Effects of tapering levothyroxine treatment in elderly subjects: a self-controlled study.
The RELEASE study

Introduction

Dear Sir/Madam,

With this letter, we would like to ask you to take part in a medical study. Participation is voluntary. If you would like to participate, written consent is required. You have received this letter because your general practitioner (GP) has selected you based on your current treatment with artificial thyroid hormone (levothyroxine).

You can take the decision whether you wish to take part in the RELEASE study based on this information sheet which will fully explain this medical study.

- Please, read this information carefully and discuss it with your relatives, friends or family.
- Ask the coordinating physician-investigator or your GP for an explanation if you have any questions.
- You can also ask the independent expert mentioned at the end of this letter for additional information.
- Further information about taking part in a medical research study can be found in the attached brochure 'Medical-scientific research'. It can be found on the government website: https://www.rijksoverheid.nl/documenten/brochures/2014/09/01/medisch-wetenschappelijk-onderzoek-algemene-informatie-voor-de-proefpersoon. You can also request a copy of this brochure from the researcher.

1. General information

This is an investigator-initiated multicentre study. This study was set up at the Department of Public Health & Primary Care Medicine at LUMC by Ms R.K.E. Poortvliet, principal investigator and general practitioner, and Ms A.J.J. Ravensberg, physician-investigator (see appendix A). The study will be conducted by general practitioners, specialists in geriatrics and medical specialists in various general practices, nursing homes and hospitals. It is expected that 360 subjects will participate. The medical ethics review committee Leiden-Den Haag-Delft (METC LDD) has approved this study. General information on research assessment can be found in the brochure 'Medical-scientific research'. The research is funded by a grant from ZonMW.

2. What is the purpose of the study?

The purpose of this study is to find out whether people aged 60 years and older who are taking the drug levothyroxine (thyroid hormone), are treated optimally and whether some of them can reduce or even stop taking levothyroxine.

3. What is the background of the study?

In the Netherlands, many people have been prescribed drugs containing artificial thyroid hormone (levothyroxine). This treatment is started in people with a marked thyroid hormone deficiency, but it

can also be started in case of a mildly reduced thyroid function. In approximately 15% of all people taking levothyroxine, it is unknown why this drug should be taken.

Levothyroxine treatment is usually continued for life, and is not always monitored regularly. However, there are several reasons why levothyroxine treatment should be monitored regularly and why its usefulness should be reassessed. With age, the body may need less levothyroxine. In addition, some of the reasons to prescribe levothyroxine in the past are now outdated according to current guidelines. For instance, new studies show that levothyroxine treatment later in life does not lead to better health for many individuals with a mildly reduced thyroid function. Moreover, laboratory tests of thyroid function have improved, making it easier today to determine whether levothyroxine treatment is necessary.

Therefore, it is not unexpected that research predicts that a proportion of levothyroxine users aged 60 and above may be treated unnecessarily and thus may not benefit from levothyroxine for their health.

What is the research question?

The RELEASE study investigates how many people aged 60 and older using long-term levothyroxine treatment can gradually discontinue the use of levothyroxine, while maintaining a normal value of thyroid hormone (free T4) and a value of the thyroid stimulating hormone (TSH) of less than 10 mU/L over a period of 1 year. In addition, effects on quality of life and general health will be studied. We will also determine which factors can predict whether levothyroxine treatment can be gradually discontinued while thyroid values remain normal.

Are you eligible to take part?

A total of 360 people aged 60 years and over who have been taking levothyroxine at a stable dose for at least 1 year can participate in this study.

When can you NOT participate in this study?

- If the thyroid gland has been removed.
- If the thyroid gland or neck has been irradiated.
- After treatment with radioactive iodine.
- If there is a thyroid disorder due to use of amiodarone or lithium.
- If you are taking the thyroid hormone liothyronine.
- If you use thiamazole, carbimazole or propylthiouracil (medication that inhibits the thyroid gland).
- If the thyroid disorder is caused by a brain disorder.
- If your last TSH value is equal to or greater than 10 mU/L.
- If your current dose of levothyroxine is higher than 150 micrograms per day
- If you are diagnosed with severe heart failure, dementia or terminal illness.
- If there is incapacity of will.

Ravensberg AJ, *et al. BMJ Open* 2023; 13:e070741. doi: 10.1136/bmjopen-2022-070741

4. What happens during the study?

How long will the study take?

Participation in this study takes about 15 months in total.

How will levothyroxine treatment be discontinued?

Your GP will gradually discontinue treatment with levothyroxine in steps, upon agreement with you. This is done partly on the basis of your thyroid function as determined from a blood sample.

Study and measurements

The study consists of a preparation phase, a control phase, a discontinuation phase, a final follow-up, and the collection of medical data.

Preparation

- If you agree to take part in the study, you will be requested to sign a consent form. The signed consent form must be sent to the LUMC research centre.
- You will then be contacted by phone by the physician-investigator or research team member to discuss the study and the completed consent form. If you decide you to participate in the study after this telephone conversation, you will continue with the control phase.

Control phase

Week 1

- You will receive a number of questionnaires at home with general questions about your health, your medication use and your well-being. Please fill these in and send them back. This will take about an hour.
- You will be requested to visit your own GP laboratory to draw blood to check your thyroid function. Preferably, you should visit the laboratory in the morning before you have had breakfast and before you have taken your medication. If necessary, the laboratory can visit you at home.
- You will be requested to discuss the results from the thyroid check with your GP and to continue your usual dose of levothyroxine for 12 weeks.
- However, if your thyroid function is abnormal (free T4 is too low; TSH is too high [≥ 10 mU/L]), you cannot participate in the study. Your GP will then determine which following steps are necessary.

Discontinuation of levothyroxine

Step 1 (week 13)

- You will be requested to complete some questionnaires (this takes about 30 minutes) and to have another blood test to determine thyroid function.
- Your GP will discuss with you whether you are eligible to start discontinuation of levothyroxine. If so, you will receive a prescription for a lower dose of levothyroxine. Collect your new levothyroxine tablets from the pharmacy (or have them delivered) and start taking them.

Step 2

Ravensberg AJ, *et al. BMJ Open* 2023; 13:e070741. doi: 10.1136/bmjopen-2022-070741

- Six weeks after the first dose-lowering step, you are requested to fill in questionnaires again (this takes about 20 minutes) and have another blood test to determine thyroid function.
- Based on this check and how you are doing, your GP will discuss with you whether levothyroxine treatment can be further tapered. If so, you will receive a new prescription and continue with lower dose of levothyroxine.

Other steps

- As long as levothyroxine treatment can be further tapered, the dose will be reduced every 6 weeks until it can finally be stopped.
- Monitoring of the thyroid function (in a blood sample) takes place after each dose-lowering step and after stopping. You will also receive questionnaires after stopping levothyroxine (completion takes about 20 minutes).
- How many dose-lowering steps you can complete in total, depends partly on how high your usual dose of levothyroxine was at the start of discontinuation. There is a maximum of 6 dose-lowering steps. The discontinuation phase is estimated to last 2 to 6 months for most participants. In extreme cases, it may take around 10 months.

Final follow-up

- One year after starting dose-lowering of levothyroxine, you will receive the last set of questionnaires to fill in (this takes about 30 minutes). Also you are requested to have a final blood test to determine your thyroid function. Your GP will discuss the results with you.

You will be asked for permission to request medical data from your GP at the start of the study for up to 2 years afterwards. This concerns data from your medical file about your medication use, your medical history, any serious illnesses and/or hospital admissions that occurred during the study period, and any side effects during the discontinuation phase.

5. What agreements do we make with you?

In order for the study to run smoothly, and for your own safety, we want to make the following agreements with you:

- You have blood tests taken to determine your thyroid function several times at your GP's laboratory (at least 4 times). This should preferably be done in the morning before you have had breakfast and before you have taken your levothyroxine. If necessary, a laboratory staff member can visit your home.
- You discuss with your GP, the results of the blood test and the possible tapering of your levothyroxine treatment. This can be done by telephone or during a visit to the general practice.
- You collect a new prescription for a lower dose of levothyroxine with each tapering off step and to start the new dose of levothyroxine as soon as possible.
- You complete 5 or 6 sets of questionnaires at home, and to return these to the research centre before the results of the latest blood sample for thyroid function are known. Completion of the questionnaires takes about 60 minutes for the first set, and 20-30 minutes for each following set.

Additional points of interest:

- This study does not impose restrictions on any of your activities.

- We collect data from your medical records from your GP for the purpose of the study (see bullet nr 4).
- We will inform your GP of your participation in this study.

It is important that you contact the research centre during the study:

- If you are hospitalised.
- If you suddenly develop health problems.
- If you no longer wish to participate in the study.
- If your contact details change.

6. What side effects, adverse effects or discomforts could you experience?

During the discontinuation phase, it is possible that the level of thyroid hormone may become too low (freeT4) or that the level of thyroid stimulation hormone (TSH) may become too high. This may cause symptoms of an inactive thyroid gland such as fatigue, intolerance of cold, muscle ache, constipation and/or a mood change. In this case, your GP will restart levothyroxine or increase the dose.

If you or your GP identify possible side effects, your GP will assess whether you can continue to lower the dose of levothyroxine or not.

7. What are the pros and cons if you take part in the study?

It is important that you carefully weigh up the possible advantages and disadvantages before you decide if you want to participate in this study.

Advantages of participating in the study

- It cannot be guaranteed that you will personally benefit from participating in this study.
- A possible benefit of participation may be that you will eventually no longer need to take levothyroxine or can continue to take a lower dose.
- Your thyroid function will be monitored regularly and your levothyroxine treatment will be reassessed by your GP.
- In the future, the RELEASE study may contribute to improved treatment of thyroid disorders in older adults.

Disadvantages of participating in the study

- It is not expected that you will experience any adverse effects from the blood collection. The total amount of blood collected at each measurement time of the study is a maximum of 10 ml (1 tube of 10 ml).
- You may possibly experience side effects from phasing out levothyroxine: see section 6.

8. What happens if you do not wish to participate or if you wish to stop taking part in the study?

It is up to you to decide whether you wish to participate in the study. Participation is voluntary. If you do not wish to take part, you will be treated with levothyroxine as usual by your GP. If you decide not to participate, no more steps need to be taken. Optionally, you can voluntarily and anonymously return our reply card with the provided envelope. On the reply card, we will ask you about the reason(s) not to take part in the study to improve future scientific research.

If you do take part, you may always change your mind and choose to stop participation, even during the study. You will then be treated as usual with levothyroxine by your GP. You do not have to give a

reason for stopping. However, you must report stopping to the researcher immediately. The data collected up to that point (including the blood test results) will be used for the study.

The investigator will let you know if there is any new information about the study that is important to you. The investigator will then ask you if you want to continue to take part.

9. When does the study end?

In these situations, the study will stop for you:

- if all the visits described in point 4 are finished,
- if you choose to stop,
- if the investigator or GP considers it better for you to stop,
- if the principal investigator, the government or the reviewing medical ethics committee decides to stop the study.

The entire study ends when all participants have finished and all data have been collected. The GP will discuss with you at the last study visit how to proceed with your medical care (15 months after the start of the study).

After processing all the data, the researcher will inform you and your GP about the main outcomes of the study. This will take place a few months after the last participant has discontinued levothyroxine for 2 years.

10. What will be done with your data?

For this study, your personal data will be used and stored. This includes data such as your name, address, date of birth and data about your health. Blood samples to determine thyroid function are required for this study. The results of these tests are used and stored by the researcher. No blood samples are kept by the researcher. The collection, use, and storage of your data is necessary to answer the research questions of this study and to publish the results. We ask for your consent to use your data.

How do we protect your privacy?

To protect your privacy, your data will be coded. Your name and other data that can directly identify you, are removed. Only the key to the code allows data to be traced back to you. The key to the code remains securely stored at the local research facility. The data sent to the researcher contain only the code, but not your name or any other data that could identify you. In reports and publications about this study, data cannot be traced back to you.

Who can see your data?

Some people can access all your data at the research site, also data without a code. This is necessary to check whether the study has been carried out properly and reliably. Persons who can access this data for control purposes are: the committee monitoring the safety of the study, a monitor of the study, a controller working for the researcher, national supervisory authorities, e.g. the Inspector for Youth and Healthcare. They will keep your data confidential. We will ask you to give permission for this inspection.

For how long do we store your data?

Your data must be kept at the research site for 15 years.

Can we use your data for other research?

After this study ends, your data may also be relevant to other scientific questions in the field of thyroid disease and levothyroxine treatment. For this purpose, we would like to keep your data for 20 years. You can indicate on the consent form whether or not you agree to this. If you do not agree to this, it is still possible to take part in the RELEASE study. In addition, we ask for your permission to approach you again in the future (after the study has ended) for possible participation in follow-up research.

Can you take back your consent for the use of your data?

You can withdraw your consent to use your personal data at any time. This applies to this study and also to storage and use for possible future research. But please note: if you take back your consent, and the investigators have already collected data for research, they are still allowed to use this information, including the results of the blood tests.

Do you want to know more about your privacy?

For general information on your rights when processing your personal data, please consult the website of the Personal Data Authority or visit https://www.lumc.nl/12367, where you will find more information on LUMC's Privacy Statement. If you have any questions about your rights, please contact the person responsible for processing your personal data. For this study, this is the researcher at the Department of Public Health and Primary Care Medicine at LUMC. See Appendix A for contact details.

If you have any questions or complaints about the processing of your personal data, we advise you to contact the research site first. You can also contact the institution's Data Protection Officer (see Appendix A) or the Personal Data Authority.

Where can you find more information about the study?

Information about this study is also included in a register of medical scientific studies namely www.trialregister.nl. This does not include data traceable to you. After the study, the website may show a summary of the results of this study. You will find this study under 'RELEASE study'.

11. Are you insured during the study?

Insurance has been taken out for everyone taking part in this study. The insurance pays for damage caused by the study. Not all damage is covered. You can find more information about this insurance and any exceptions in Appendix B. It also says who you can report damage to.

12. We will inform your general practitioner

We will always send your GP a message to inform them that you are participating in the study. This is for your own safety. If you do not agree, you cannot take part in this study. Your GP will be informed as soon as you have given consent to participate. The GP has an important role in lowering the dose of levothyroxine and signaling any side effects.

13. Will you receive compensation if you participate in the study?

The additional checks of thyroid function in blood will be reimbursed. You will receive a reimbursement of € 18.50 per blood sample. These checks and contacts with the GP will not affect

your deductible excess. Transport costs for visits to the GP, pharmacy and/or GP laboratory will not be reimbursed.

14. Do you have any questions?

If you have any questions, please contact your own GP or the coordinating research physician, Ms A.J.J. Ravensberg (LUMC, during office hours, telephone +31 71 526 86 40). You can also send an email to release@lumc.nl. Would you like to get advice from someone who is independent from the study? Then contact the independent expert Ms Dr P.G. van Peet, who can be reached at p.g.van_Peet@lumc.nl or by phone at +31 71 526 844. She is a GP and is aware of all the details of the study, but is not part of this study.

Do you have a complaint? Discuss it with the investigator or the doctor who is treating you. If you prefer not to do so, please visit please contact the LUMC patient service office. Appendix A tells you where to find this.

15. How do you give consent for the study?

You can first think carefully about this study for a period of 4 weeks. Then you tell the investigator if you understand the information and if you want to take part or not. If you want to take part, fill in the consent form that you can find with this information sheet. Your written consent indicates that you have understood the information and agree to participation in the study. You and the investigator will both get a signed version of this consent form.

Thank you for your attention.

16. Appendices to this information

A. Contact details

B. Insurance information

C. Consent forms

Brochure 'RELEASE - Thyroid hormone treatment in people over sixty, a closer look'

A. Contact details RELEASE study

Principal investigator

Ms Dr R.K.E. Poortvliet

Senior general practitioner researcher LUMC

E-mail: r.k.e.poortvliet@lumc.nl

Telephone (during office hours): +31 71 526 8640

Coordinating researcher and first point of contact

Ms A.J.J. Ravensberg

Research physician

E-mail: release@lumc.nl

Telephone (during office hours): +31 71 526 8640

Independent physician

Mrs Dr P.G. van Peet

GP

E-mail: p.g.van_peet@lumc.nl

Telephone (during office hours): +31 71 526 84 44

Complaints

If you do not wish to contact the research team directly, you can always contact the LUMC patient service office.

E-mail: patientenservicebureau@lumc.nl

Telephone (Mon to Fri from 08.30 - 16.30): +31 71 526 2989

Data protection officer of the institution

E-mail: infoavg@lumc.nl

For more information on your rights:

https://www.lumc.nl/12367

B. INFORMATION TEST SUBJECT INSURANCE

For scientific research with risks in and/or by LUMC

'Effects of tapering treatment with levothyroxine in elderly subjects: a self-controlled study - The RELEASE study'

Protocol number: NL69753.058.19

The LUMC has taken out insurance for everyone who takes part in the study. The insurance pays for any damage you might suffer as a result of participation in this study. This concerns damage due to death or injury, which reveals itself during participation in this research and damage that reveals itself within four years after participation in this research.

The amount covered by the insurance is:

1) €650,000 for damages per subject,

2) €5,000,000 for the damage of all subjects together participating in this study

3) €7,500,000.00 for the total damage manifested per insurance year to test subjects in all investigations the client commissions per insurance year.

Excluded from coverage by this insurance is:

- damage resulting from failure to improve or deterioration of the subject's health problems, if participation in the research is in the context of treatment of these health problems;
- damage due to impairment of the subject's health which is likely to have manifested itself even if the subject had not participated in this investigation.
- damage due to impairment of the subject's health if such damage results from participation in a scientific study consisting of a comparison of two or more usual treatments or procedures;
- damage manifesting itself in offspring as a result of an adverse effect of the research on the subject and/or the offspring;
- damage of which it is certain or virtually certain by the nature of the research that it would occur in the subject;
- damage resulting from the subject's failure to comply or not fully comply with directions and instructions, insofar as the subject is capable of doing so;
- damage resulting from the occurrence of a risk for which you were warned in the written information, unless the risk occurs to a greater extent than was foreseen or the risk was extremely unlikely.

The name of the insurance company where the study is insured is:

Onderlinge Waarborgmaatschappij Centramed B.A.
Appelgaarde 4
2272 TK Voorburg

If one has suffered or suspects damage, one should contact the investigator or the treating physician as soon as possible.

Ravensberg AJ, *et al. BMJ Open* 2023; 13:e070741. doi: 10.1136/bmjopen-2022-070741

C. Subject consent form 'Effects of tapering treatment with levothyroxine in elderly subjects: a self-controlled study - The RELEASE study'

- I have read the information letter. I was also able to ask questions. My questions have been answered well enough. I had enough time to decide if I wanted to take part.
- I know that taking part is voluntary. I also know that at any time I can decide not to take part in the study, or to stop taking part. I do not have to explain why.
- I give the investigator consent to inform my treating physician (GP, geriatric specialist or hospital doctor) that I am taking part in this study.
- I give consent to request medical information from my treating physician for the purpose of the study.
- I give consent to collect and use my data and the results of my blood tests for answering the research question in this study.
- I know that some people will be able to see all my data to review the study. These people are listed in this information letter. I give consent to let them see my data for this review.
- I give consent to keep my research data for 15 years after the end of this study.
- I do □ / do not □
  give consent to keep and use my personal data longer for future research in the field of thyroid disorders and levothyroxine treatment, namely for the duration of 20 years. *
- I do □ / do not □
  give consent to the investigator to contact me for a follow-up study after this study. *
- I want to take part in this study.

* Check the relevant box

Data subject

Name Mr/Mrs .... Date of birth ....

Address .... Postal code.......................................

City .... Phone number ....

Signature .... Date ....

____

To be completed by the research officer of RELEASE study

I declare that I have fully informed this subject about the study mentioned.

If any information becomes known during the study that could influence the subject's consent, I will let this subject know in good time.

Investigator name (or their representative):

Signature: Date: __ / __ / __

Additional information was given by (if applicable):

Name:

Job title:

Signature: Date: __ / __ / __

The study subject will receive a complete information sheet, together with a signed version of the consent form.

Ravensberg AJ, *et al. BMJ Open* 2023; 13:e070741. doi: 10.1136/bmjopen-2022-070741
