# Supplementary material for: Effects of discontinuation of levothyroxine treatment in older adults: protocol for a self-controlled trial
Source: BMJ Open. 2023 Apr 25;13(4):e070741. doi: 10.1136/bmjopen-2022-070741 (PMC10151847; doi:10.1136/bmjopen-2022-070741)
Supplement: Supplementary data [file bmjopen-2022-070741supp002.pdf]

Appendix 2 – Outcomes

Instruments used to determine baseline characteristics and secondary outcome parameters

Integrated Systematic Care for Older Persons (ISCOPE) Questionnaire

The ISCOPE questionnaire indicates the degree of complex problems in older participants by measuring problems in the functional, somatic, psychological and social domain as experienced by older persons (1, 2). This questionnaire consists of 26 closed questions. The score ranges from 0 (no problems in those domains) to 4 (problems on 4 domains).

Revised Patients' Attitudes Towards Deprescribing (rPATD) questionnaire

The revised Patients 'Attitudes Towards Deprescribing (rPATD) questionnaire was developed to capture older adults' and caregivers' beliefs and attitudes towards deprescribing (3). The rPATD was developed and validated for older adults ≥65 years of age taking at least one regular medication and caregivers of older adults. The revised PATD questionnaire had acceptable validity and reliability.

Treatment satisfaction (TSQM)

The Treatment Satisfaction Questionnaire for Medication version II (TSQM) was developed as a measure of treatment satisfaction for medication without disease-specific focus (4). This allows the questionnaire to be well fitted to a range of participant conditions and characteristics. Furthermore it allows for sound comparing of treatment satisfaction with other medication or medication discontinuation studies. The TSQM has been validated in multiple languages (including Dutch) and is reported to have sound psychometric properties. It consists of 11 questions, all using 7-point Likert scales.

Goal Attainment Scale (GAS)

The GAS questionnaire will be used to ask participants what dose reduction of levothyroxine treatment they would define as a substantial dose reduction (at pre-baseline), and whether they achieved this (at final follow-up).

Thyroid-specific quality of life (ThyPro39)

The ThyPRO39 is a questionnaire covering a comprehensive range of thyroid-related quality of life issues and considerations spanning somatic, psychological and social domains. It consists of 84 questions, all 5-point Likert scale, ranging 13 thyroid-specific quality of life domains. In a systematic review the ThyPRO was nominated as the recommended method for assessing health-related quality of life in benign thyroid diseases (5). Measuring ThyPRO39 at baseline, 6 weeks after the start of the

discontinuation phase and at the end of the discontinuation phase, will give insight to symptoms that may have triggered stopping the discontinuation phase.

Quality of life (EQ5D)

The EQ5D questionnaire is a standardized instrument for use as a measure of health outcome (6). The Visual Analogue Scale is a horizontal line ranging from 0 (worst imaginable health) to 100 (perfect health) on which the patients mark the point that they feel represents their perception of their current health state.

Decision Regret Scale (DRS)

The Decision Regret Scale is a validated indicator of health care decision regret at a given point in time (7). It will be used at the final follow-up to evaluate regret after the decision to withdraw levothyroxine treatment. The questionnaire consists of 5 items. The participant indicates for each statement the extent to which they agree or disagree. Scoring consists of reversing the scores of the 2 negatively phrased items (2 and 4), then taking the mean of the 5 items. These means can be converted to a score ranging from 0 to 100 by subtracting 1 and multiplying by 25.

References

1. Blom J, den Elzen W, van Houwelingen AH, Heijmans M, Stijnen T, Van den Hout W, et al. Effectiveness and cost-effectiveness of a proactive, goal-oriented, integrated care model in general practice for older people. A cluster randomised controlled trial: Integrated Systematic Care for older People--the ISCOPE study. Age Ageing. 2016;45(1):30-41.
2. van Houwelingen AH, den Elzen WP, le Cessie S, Blom JW, Gussekloo J. Consequences of interaction of functional, somatic, mental and social problems in community-dwelling older people. PLoS One. 2015;10(4):e0121013.
3. Reeve E, Low LF, Shakib S, Hilmer SN. Development and Validation of the Revised Patients' Attitudes Towards Deprescribing (rPATD) Questionnaire: Versions for Older Adults and Caregivers. Drugs Aging. 2016;33(12):913-28.
4. Atkinson MJ, Kumar R, Cappelleri JC, Hass SL. Hierarchical construct validity of the treatment satisfaction questionnaire for medication (TSQM version II) among outpatient pharmacy consumers. Value Health. 2005;8 Suppl 1:S9-s24.
5. Wong CK, Lang BH, Lam CL. A systematic review of quality of thyroid-specific health-related quality-of-life instruments recommends ThyPRO for patients with benign thyroid diseases. J Clin Epidemiol. 2016;78:63-72.

6. EuroQol. EuroQol--a new facility for the measurement of health-related quality of life. Health Policy. 1990;16(3):199-208.

7. Brehaut JC, O'Connor AM, Wood TJ, Hack TF, Siminoff L, Gordon E, et al. Validation of a decision regret scale. Med Decis Making. 2003;23(4):281-92.
