# Supplementary material for: Effects of discontinuation of levothyroxine treatment in older adults: protocol for a self-controlled trial
Source: BMJ Open. 2023 Apr 25;13(4):e070741. doi: 10.1136/bmjopen-2022-070741 (PMC10151847; doi:10.1136/bmjopen-2022-070741)
Supplement: Supplementary data [file bmjopen-2022-070741supp003.pdf]

Appendix 3. Trial information

Trial registration data

**Data category**	**Information**
Primary registry and trial identifying number	Netherlands Trial Register NL7978
Date of registration in primary registry	22 August, 2019
Secondary identifying numbers	ZonMW projectnumber 839110026
Source(s) of monetary or material support	ZonMW HGOG
Primary sponsor	Leiden University Medical Center
Secondary sponsor(s)	Not applicable
Contact for public queries	R.K.E. Poortvliet, MD, PhD – Leiden University Medical Center – release@lumc.nl
Contact for scientific queries	R.K.E. Poortvliet, MD, PhD – Leiden University Medical Center – r.k.e.poortvliet@lumc.nl
Public title	REduction of LEvothyroxine in Adults; a SElf-controlled study (RELEASE)
Scientific title	Effect of discontinuation of levothyroxine treatment in older adults; a self-controlled study
Countries of recruitment	The Netherlands
Health condition(s) or problem(s) studied	Hypothyroidism
Intervention(s)	Stepwise reduction of levothyroxine treatment
Key inclusion and exclusion criteria	Inclusion criteria:<br>1) Aged 60 years or over (= 60 years)<br>2) Using any levothyroxine mono-therapy medicament (ATC: H03AA01) continuously for a minimum of 1 year with stable dose of levothyroxine.<br>Exclusion criteria:<br>1) Last measurement of TSH =10 mU/L during levothyroxine treatment<br>2) Current reason for levothyroxine treatment: patients with history of thyroidectomy; radioactive iodine treatment or neck irradiation; congenital hypothyroidism; secondary hypothyroidism, or concurrent amiodarone or lithium use<br>3) Dose of treatment; for safety issues, patients using > 150 mcg levothyroxine per day will not be eligible<br>4) Diagnosis of heart failure NYHA grade IV<br>5) Participation in ongoing trials of therapeutic interventions<br>6) Life-expectancy of less than 6 months<br>7) Diagnosis of dementia<br>8) Incapacitated adults<br>9) Persons that plan to move out of the region

	in which the study is being conducted in the next months.
Study type	Observational
Date of first enrolment	13-02-2020*
Target sample size	385*
Recruitment status	Active, not recruiting*
Primary outcome(s)	The proportion of participants that withdraw their thyroid medication successfully defined as normal fT4 levels and TSH levels <10 mU/L at 52 weeks after the start of the discontinuation.
Key secondary outcome(s)	1. The proportion of participants that achieve a substantial dose reduction of levothyroxine at 52 weeks a) defined as ≥50% and b) defined by the participants themselves; 2. The effect of discontinuation of levothyroxine treatment at 52 weeks on a) thyroid-related quality of life and b) general health; 3. How participants reflect on their decision to discontinue levothyroxine treatment.

* These are updated data. The Netherlands Trial Register is no longer available. Trial data registered in the NTR can be consulted on the International Clinical Trial Registry Platform (ICTRP) Search Portal, but it is no longer possible to adjust these data. This study will be re-registered at ClinicalTrials.gov to keep an up to date trial registration.

Trial sponsor

Leiden University Medical Center

Sponsor's reference: NL69753.058.19

Contact name: Prof. dr. J. Gussekloo

Address: Leiden University Medical Center, Department of Public Health and Primary Care

Postzone V-0-P; PO BOX 9600; 2300 RC Leiden, the Netherlands

Telephone: +31 715268444

Email: j.gussekloo@lumc.nl

Roles and responsibilities – committees

Principal investigators: J. Gussekloo, R.K.E. Poortvliet, R.S. Du Puy

- Responsible for the design and conduct of the study

Coordinating investigator /project leader: R.K.E. Poortvliet and Research physician: A.J. Ravensberg

- Preparation of protocol and revisions
- Preparation of investigators brochure (IB) and case report forms (CRFs)
- Organising steering committee meetings
- Publication of study reports

Steering committee (SC): J. Gussekloo, R.K.E. Poortvliet, R.S. Du Puy; A.J. Ravensberg; O.M. Dekkers; S.P. Mooijaart

- Agreement of final protocol
- Reviewing progress of study and if necessary agreeing changes to the protocol and/or investigators brochure to facilitate the smooth running of the study.

Trial management committee (TMC)

- Members: coordinating investigator R.K.E. Poortvliet and research physician A.J. Ravensberg
- Study planning
- Organization of SC meetings
- Establish regular reports for the Institutional Ethics Committee and the Data Safety Monitoring Board (DSMB)
- SAE [serious adverse event] and SUSAR [serious unexpected suspected adverse events] reporting to the Dutch Central Committee on Research Involving Human Subjects (CCMO) and to the DSMB
- Responsible for trial master file
- Budget administration and contractual issues with individual centers (general practices)
- Advice for lead investigators
- Preparing and follow-up of trial audits
- Assistance with international review, board/independent ethics committee applications
- Data verification
- Organisation of data collection

Data management: A.J. Ravensberg; B.M. Schakenbos; H. de Jong

- Maintenance of trial IT system and data entry
- Data verification
