# Supplementary material for: Effects of discontinuation of levothyroxine treatment in older adults: protocol for a self-controlled trial
Source: BMJ Open. 2023 Apr 25;13(4):e070741. doi: 10.1136/bmjopen-2022-070741 (PMC10151847; doi:10.1136/bmjopen-2022-070741)
Supplement: Author's manuscript [file bmjopen-2022-070741.draft_revisions.pdf]

BMJ Open

BMJ Open is committed to open peer review. As part of this commitment we make the peer review history of every article we publish publicly available.

When an article is published we post the peer reviewers' comments and the authors' responses online. We also post the versions of the paper that were used during peer review. These are the versions that the peer review comments apply to.

The versions of the paper that follow are the versions that were submitted during the peer review process. They are not the versions of record or the final published versions. They should not be cited or distributed as the published version of this manuscript.

BMJ Open is an open access journal and the full, final, typeset and author-corrected version of record of the manuscript is available on our site with no access controls, subscription charges or pay-per-view fees (http://bmjopen.bmj.com).

If you have any questions on BMJ Open's open peer review process please email info.bmjopen@bmj.com

BMJ Open

Study protocol: the effects of discontinuation of levothyroxine treatment in older adults - a self-controlled trial

Journal:	*BMJ Open*
Manuscript ID	bmjopen-2022-070741
Article Type:	Protocol
Date Submitted by the Author:	02-Dec-2022
Complete List of Authors:	Ravensberg, A.; Leiden University Medical Center, Department of Public Health and Primary Care<br>Poortvliet, Rosalinde K. E.; Leiden University Medical Center, Department of Public Health and Primary Care; Leiden University Medical Center, University Network for the Care sector Zuid-Holland<br>Du Puy, Robert ; Leiden University Medical Center, Department of Public Health and Primary Care<br>Dekkers, Olaf; Leiden University Medical Center, Department of Endocrinology and Metabolic Disorders<br>Mooijaart, Simon; Leiden University Medical Center, Department of Gerontology and Geriatrics<br>Gussekloo, Jacobijn; Leiden University Medical Center, Department of Public Health and Primary Care; Leiden University Medical Center, Department of Gerontology and Geriatrics
Keywords:	Thyroid disease < DIABETES & ENDOCRINOLOGY, Quality of Life, GENERAL MEDICINE (see Internal Medicine), PRIMARY CARE, GERIATRIC MEDICINE

SCHOLARONE™
Manuscripts

TITLE

Study protocol: the effects of discontinuation of levothyroxine treatment in older adults - a self-controlled trial

Authors

A. Janneke Ravensberg[1]

R.K.E. Poortvliet[1, 2]

R.S. Du Puy[1]

O.M. Dekkers[3]

S.P. Mooijaart[4]

J.G. Gussekloo[1]

[1]Department of Public Health and Primary Care, Leiden University Medical Center, Leiden, the Netherlands.

[2] University Network for the Care sector Zuid-Holland, Leiden University Medical Center, Leiden, the Netherlands

[3]Department of Endocrinology and Metabolic Disorders, Leiden University Medical Center, Leiden, the Netherlands.

[4]Department of Gerontology and Geriatrics, Leiden University Medical Center, Leiden, the Netherlands.

Corresponding author:

Janneke Ravensberg

Leiden University Medical Center, Department of Public Health and Primary Care

Postzone V-0-P; PO BOX 9600; 2300 RC Leiden, the Netherlands

a.j.j.ravensberg@lumc.nl

tel: +31 71 526 8444; fax: +31 71 526 8259

Wordcount: 3175

ABSTRACT

Background Many older persons use the thyroid hormone levothyroxine which is often continued for life. Scientifically, there is much uncertainty whether simple continuation is the optimal approach. First, the physical need for levothyroxine can decrease with age thereby posing a higher risk of overtreatment and adverse effects. Second, large trials in subclinical hypothyroidism have shown no benefit for the use of levothyroxine. Interestingly, guidelines do not address re-evaluation of the indication. This self-controlled trial aims to determine the effects of discontinuation of levothyroxine treatment in older adults.

Methods and analysis Participants are community-dwelling subjects aged ≥ 60 years using levothyroxine continuously at a stable dosage of ≤ 150 microgram and a level of TSH <10 mU/L. After a control period of 12 weeks, levothyroxine treatment is discontinued gradually using a step-wise approach with regular monitoring of thyroid function guided by their general practitioner. The primary outcome is the proportion of participants withdrawn from levothyroxine while maintaining a free T4 level within the reference range and a TSH level <10 mU/L, 52 weeks after the start of discontinuation. Secondary outcomes are compared to the control period (self-controlled) and include amongst others, the effects on thyroid-specific and general health-related quality of life. Furthermore, patients' attitudes towards deprescribing and regret regarding discontinuing levothyroxine treatment will be recorded. A total of 518 participants will be recruited to estimate the expected proportion of 50% with a 95% confidence interval ranging from 45% to 55%.

Ethics and dissemination Approval was obtained from the institutional medical ethics committee. The Older People Advisory Board Health and Wellbeing has reviewed the research proposal and their comments were used for improvement. In line with the funding policies of the grant organisation funding this study, the study results will be proactively disseminated to the general public and key public health stakeholders.

Trial registration Netherlands Trial Register; NL7978; registered 22 August 2019.

Protocol version version 6.0; 20 September 2022.

For peer review only - http://bmjopen.bmj.com/site/about/guidelines.xhtml

Keywords (MeSH descriptors)

1. Humans
2. Aged;
3. Aged, 80 and over;
4. Thyroxine;
5. Deprescriptions;
6. Observational Study;
7. Thyroid Diseases;
8. Hypothyroidism;
9. Quality of Life;
10. Health Status;
11. Personal Satisfaction;
12. General Practitioners

Strengths and limitations of this study

- This is a self-controlled trial to assess the effect of discontinuation of levothyroxine treatment specifically in older adults (aged ≥ 60 years), using both thyroid function and thyroid-related quality of life as outcome parameters.
- Stepwise dose reduction with regular measurements of thyroid function reduces the risk of prolonged periods of undertreatment potentially resulting in symptoms of hypothyroidism and is easily implementable to apply in clinical practice.
- This study is not blinded which may introduce performance bias and detection bias. However, the primary endpoint, successful withdrawal, is a biochemically defined endpoint, which is not affected by the unblinded design of the study. If successful discontinuation of levothyroxine can be achieved in this study, this concept may be improved in daily practice.

For peer review only - http://bmjopen.bmj.com/site/about/guidelines.xhtml

INTRODUCTION

Levothyroxine is one of the most widely prescribed drugs and is currently ranked in the top 3 of total prescriptions in the United States (US) and United Kingdom (UK) (1, 2). It is most frequently prescribed and initiated in older adults (aged 50-70 years) (3). Notably, levothyroxine prescriptions have increased substantially, from 2.3% to 3.5% over the past decades despite a low prevalence (0.1-1.9%) and a stable incidence of overt hypothyroidism (2, 4). A contributing factor to the rise in levothyroxine prescriptions is likely an increase in the treatment of subclinical hypothyroidism (5).

Subclinical hypothyroidism is a common condition defined by an elevated level of thyroid-stimulating hormone (TSH) and a free T4 (fT4) level within the normal reference range. The prevalence of subclinical hypothyroidism increases with age (5-20% in men and women older than 60 years) (6). A recent US study showed that most individuals who started on levothyroxine were diagnosed with subclinical hypothyroidism (65.7%) (7). Furthermore, studies indicate that TSH levels at the initiation of treatment are falling (8, 9), which is worrisome because it may increase the chance of ineffective treatment and the risk of overtreatment (10). This holds especially for older adults with subclinical hypothyroidism with TSH levels <10 mU/L since recent randomized clinical trials show that levothyroxine treatment does not improve thyroid-related symptoms, quality of life, depressive symptoms, or cognitive function in older people (11-13).

Treatment with levothyroxine is often continued for life, as current guidelines do not advise re-evaluation of the indication and effect (8, 14). Initial indications for treatment are not always well documented and can be inaccurate or even inappropriate (15). Patients may have started levothyroxine in the past for a diagnosis made during transient hypothyroidism (15-17). Furthermore, thyroid (dys-)function varies over time (18), and thyroid metabolism changes with age (19). Therefore, it is likely that a considerable proportion of older adults are treated with levothyroxine without a clear indication according to current guidelines when re-evaluating. This could be harmful, considering that overtreatment occurs in up to 40% of levothyroxine users (8, 20, 21) and increases the risk of bone fractures and atrial fibrillation (22, 23). The risk of overtreatment increases with treatment duration (8) and although guidelines warrant careful medication monitoring, levothyroxine dosages may remain unchanged for a long time (24).

Taking into account the high levothyroxine use in older age, the controversy regarding treatment of subclinical hypothyroidism, the lack of re-evaluation of indications, and the health risks associated with overtreatment, it can be hypothesized that discontinuation of levothyroxine treatment can be achieved without negative consequences for a considerable proportion of older adults. A meta-analysis based on observational studies including participants aged 2-81 years suggested that approximately 37% remain euthyroid after thyroid hormone discontinuation (25).

For peer review only - http://bmjopen.bmj.com/site/about/guidelines.xhtml

However, the evidence was of low quality, the adult participants were mostly middle-aged, and no study followed a systematic approach for deprescribing levothyroxine.

Therefore, the primary aim of this self-controlled trial is to investigate what proportion of community-dwelling levothyroxine users aged 60 years and older can withdraw from levothyroxine treatment successfully. Secondary aims are: 1. to study what proportion of participants can achieve a substantial dose reduction of levothyroxine at 52 weeks a) defined as ≥50% and b) defined by the participants themselves; 2. to study the effect of discontinuation of levothyroxine treatment at 52 weeks on a) thyroid-related quality of life and b) general health; 3. to determine how participants reflect on their decision to discontinue levothyroxine treatment.

For peer review only

METHODS

Study design

This is a self-controlled trial investigating the stepwise discontinuation of levothyroxine treatment in adults aged 60 years and older with a follow-up of 1 year. Outcomes will be measured 12 weeks before the start of discontinuation, at the start of discontinuation, at 6 weeks, at the end of discontinuation, and after 52 weeks. The outcomes will be compared within participants. In this way, the research questions can be studied validly in a self-controlled design, without the need for an extra control group. The formal role of a control group in a randomized trial is to estimate what would have happened in the experimental arm in the absence of the intervention (26). This effect is immediately clear in our study: levothyroxine treatment is simply continued in almost all patients. Therefore, a control group in our study will only reveal information that is already known (27). The primary outcome of the study is based on a biochemically defined thyroid function status, an fT4 level within the reference range and a TSH level <10 mU/L. Since this biochemically defined outcome is independent of participants' views or experiences, is not influenced by personal interpretation, and is therefore not affected by an unblinded design, we considered a control group as not required. A TSH level of <10 mU/L was based on the results from the recently published randomized clinical trials on levothyroxine treatment in older adults with subclinical hypothyroidism (11-13).

Study setting

Community-dwelling participants are recruited from general practitioner (GP) practices in the Netherlands. Participating GPs identify eligible patients from electronic medical records using the in- and exclusion criteria mentioned below. Selected patients receive an invitation to participate in the study and an information leaflet from their GP. Written informed consent is obtained from all patients who are willing to participate in the study.

Study population

A participant must meet all of the following inclusion criteria:

1. Age ≥ 60 years;
2. Using any levothyroxine mono-therapy medicament (ATC: H03AA01) at a stable dosage for ≥ 1 year.

Exclusion criteria are:

1. Last measurement of TSH ≥10 mU/L during levothyroxine treatment;
2. The dose of treatment is > 150 mcg levothyroxine per day;

For peer review only - http://bmjopen.bmj.com/site/about/guidelines.xhtml

3. The current reason for levothyroxine treatment: patients with a history of thyroidectomy; radioactive iodine treatment or neck irradiation; congenital hypothyroidism; secondary hypothyroidism, or concurrent amiodarone or lithium use;
4. Concurrent treatment with liothyronine (the synthetic form of thyroid hormone T3), thiamazole, carbimazole, or propylthiouracil (inhibitors of T3 and T4 synthesis);
5. Diagnosis of heart failure NYHA grade IV;
6. Participation in ongoing trials of therapeutic interventions;
7. Life-expectancy < 6 months;
8. Diagnosis of dementia;
9. Incapacitated adults;
10. Persons planning to move out of the region in which the study is being conducted in the next months.

Study phases and interventions

This study consists of 3 phases: a control phase of 12 weeks (T-12 to T0), the discontinuation phase (minimum of 6 weeks, maximum of 36 weeks; T0 to Tx), and a follow-up phase (from the end of discontinuation till 52 weeks after the start of discontinuation; Tx to T52) (see Figure 1).

Control phase Participants enter the study during a control period with pre-baseline measurements of thyroid function and secondary outcome parameters to determine natural variation after which treatment with levothyroxine is continued per usual care for at least 12 weeks (see Table 1). Participants with a pre-baseline TSH level ≥10 mU/L do not proceed to the discontinuation phase (exclusion). When a participant presents with a suppressed TSH (indicative of oversuppletion with levothyroxine), the discontinuation phase can be started immediately. Participants are requested to complete their questionnaires prior to thyroid function assessment so the results do not influence their answers. Furthermore, they are instructed to schedule all laboratory visits at the same time preferably in the morning before ingesting levothyroxine and consumption of food and/or drinks.

Discontinuation phase The discontinuation phase starts with baseline measurements of thyroid function and secondary outcome parameters (see Table 1), followed by a consultation of the participant with their GP during which discontinuation of levothyroxine is initiated, except when TSH level ≥10 mU/L. Levothyroxine is discontinued stepwise, and at intervals of ≥ 6 weeks to ascertain a steady-state concentration has been reached before the following control visit. This is in line with standard clinical practice. During each '6-week' visit, the GP evaluates thyroid function and symptoms with the participant. Upon agreement, the daily dose of levothyroxine can then be lowered provided thyroid function is within the predefined limits, i.e. a normal fT4 and TSH < 10 mU/L. With each step,

For peer review only - http://bmjopen.bmj.com/site/about/guidelines.xhtml

the dose of levothyroxine is reduced by approximately 25 mcg (the use of whole tablets is preferred) until a daily dose of 25 mcg or less is reached. The GP is free to adjust the size of the dose reduction if necessary. The maximum dose reduction in medication is 50 mcg in the first step. In the final step, the medication is discontinued completely if the laboratory results are still within the predefined limits. The discontinuation phase ends after the evaluation of complete withdrawal of the levothyroxine treatment (i.e. 6 weeks after the final discontinuation step). Secondary outcome parameters are collected at the first '6-week' visit and the end of discontinuation (see Table 1).

Participants are requested to stop the discontinuation phase when a TSH level ≥ 10 mU/L or an fT4 level below the normal range is identified during a control visit and is confirmed by an extra thyroid function measurement within two weeks. When the extra measurement does not confirm a TSH level ≥ 10 mU/L or a low fT4 level, the participant is allowed to proceed with the discontinuation phase. Other factors than thyroid function, such as symptoms, may however be a reason to prematurely stop the discontinuation or restart levothyroxine. Upon stopping the discontinuation phase, it is at the discretion of the physician and the participant to decide whether the dose of levothyroxine at that moment will be maintained or increased. Participants who have stopped the discontinuation phase prematurely will be followed up in accordance with the protocol if possible.

Follow-up phase The follow-up phase ends with a final visit 52 weeks after the start of the discontinuation phase and includes the assessment of thyroid function, current dose of levothyroxine, and secondary outcome parameters (see Table 1).

Descriptive study data and outcome measures

Descriptive data recorded at pre-baseline include:

1. Age and sex.
2. Lifestyle: smoking, alcohol intake.
3. Medical history.
4. Complex health problems (Integrated Systematic Care for Older Persons [ISCOPE] questionnaire) (28, 29).
5. The participant's attitude towards deprescribing (revised Patients' Attitudes Towards Deprescribing [rPATD] questionnaire) (30).
6. Treatment satisfaction for levothyroxine treatment (Treatment Satisfaction Questionnaire for Medication [TSQM]) (31).
7. The participant's view on what is a substantial dose reduction of levothyroxine (Goal Attainment Score [GAS]).

For peer review only - http://bmjopen.bmj.com/site/about/guidelines.xhtml

The primary outcome is the proportion of participants that withdraw their thyroid medication successfully (defined as normal fT4 levels and TSH levels <10 mU/L) at 52 weeks after the start of the discontinuation (final follow-up).

Secondary outcome measures determined at baseline and/or final follow-up include:

- Thyroid-related quality of life (ThyPRO-39) (32, 33).
- General health-related quality of life (EuroQol-5D, including the EuroQol visual analogue scale (34). Cognitive function is determined as an additional dimension (EuroQol-5D+C) (35, 36).
- Participant's view on whether they achieved a substantial dose reduction of levothyroxine (Goal Attainment Scale [GAS]).
- Participant's view on regret after the decision to withdraw levothyroxine (Decision Regret Scale [DRS]) (37).

To further explore the quality of life during the discontinuation phase, ThyPRO-39 and EuroQol measurements were also determined 6 weeks after the first step of lowering the dose of levothyroxine (T6) and 6 weeks after the last step of dose-lowering (see Table 1).

For peer review only

For peer review only - http://bmjopen.bmj.com/site/about/guidelines.xhtml

Table 1. Schedule of measurements

Time (weeks)	Study phase	Primary outcome	Secondary outcomes					Descriptives			
		TSH - FT4	ThyPro-39	TSQM	EQ5D VAS	DRS	GAS[a]	Demographics - Clinical characteristics	ISCOPE	rPATD	GAS[a]
-12	Control phase	X	X	X	X			X	X	X	X
	Discontinuation phase										
0	- start of step 1	X	X	X	X					X	
6	- control of step 1	X	X		X						
12, 18, 24, 30	- control of steps 2, 3, 4, 5	X[b]									
6/12/18/24/30/36	- control of last step[c]	X	X		X						
52	Final follow-up	X	X		X	X	X		X		

Abbreviation: TSH, Thyroid Stimulating Hormone; FT4, Free T4; ThyPRO, Thyroid-Related Quality of Life Patient-Reported Outcome; TSQM, Treatment Satisfaction Questionnaire for Medication; EQ5D, EuroQol 5 Dimensions; VAS, Visual Analogue Scale; DRS, Decision Regret Scale; GAS, Goal Attainment Scale; ISCOPE, Integrated Systematic Care for Older Persons; rPATD, revised Patients' Attitudes Towards Deprescribing.

[a] Substantial dose reduction of levothyroxine; [b] If applicable; [c] End of the discontinuation phase.

For peer review only

Sample size calculation

The sample size calculation is based on the primary outcome, the proportion of participants successfully stopping levothyroxine treatment between baseline and final follow-up at 52 weeks. Since population proportions of successful levothyroxine treatment cessation are unknown (the aim of this study), we estimated a proportion of 50% of the participants that successfully discontinue levothyroxine treatment. An estimated proportion of 50% results in the most conservative estimate (resulting in the highest calculated sample size) and is in line with the limited available evidence (39-41). We have chosen a sample size of 385 participants because this allows us to estimate the expected proportion of 50% with a 95% confidence interval of which the lower and upper limit is expected to deviate by 5 percentage points from the found percentage (i.e. 45% to 55% when the found percentage is indeed 50%).

Taking a maximum of 25% loss to follow up into account over the duration of 15 months (exclusion at T-12 when TSH level ≥ 10 mU/L and/or fT4 below reference range, withdrawal, moving away, or death between T-12 and T52) a maximum of 513 participants will be recruited.

Data analysis plan

GPs transfer participants' data after each visit using a validated electronic data capture (EDC) system (Castor EDC). Questionnaires are completed on paper or electronically (Castor EDC). All participant data are coded. Only the principal investigators and the research team have access to the key to these codes. The handling of personal data complies with the European General Data Protection Regulation. Database validation checks are run routinely and are tracked and escalated as appropriate.

To determine the proportion of the population that can be withdrawn from levothyroxine treatment, categorical data involving measurements at baseline and final follow-up will be analyzed. Univariable and multivariable logistic regression analyses will be performed to assess factors that are associated with successful discontinuation of levothyroxine treatment. To estimate the effect of discontinuation on secondary outcome parameters measured at baseline and/or final follow-up, descriptive, univariable and multivariable analyses will be performed for the overall group and for the group of participants who successfully discontinued levothyroxine. Measurements pre and post discontinuation will be compared. Variations in thyroid function, thyroid-related quality of life, treatment satisfaction for levothyroxine, general health, and participant's attitude toward deprescribing will be determined using pre-baseline and baseline measurements. All analyses will be carried out with the population of participants who enrolled at baseline (T0). Participants that experience intercurrent events not related to the study procedure resulting in missing data at final follow-up will be excluded. Primary and secondary analyses will be repeated with all participants who

For peer review only - http://bmjopen.bmj.com/site/about/guidelines.xhtml

enrolled at baseline who have data for the outcome variable of interest and have had their final follow-up visit. Statistical significance for the primary outcome requires $P \leq 0.05$.

Safety and Monitoring

Withdrawal

Participants can withdraw their consent for involvement in the study at any time, without an obligation to justify the decision, and without consequences for their usual care. The research team can advise withdrawing participants from the intervention if it is deemed to be in the best interest of the participant. Participants who withdraw from the study are not replaced. The Data Safety and Monitoring Board (DSMB) can advise to stop the study.

Serious adverse events

To avoid prolonged periods of thyroid hormone underreplacement potentially resulting in symptoms of hypothyroidism, levothyroxine treatment is discontinued gradually while monitoring thyroid function after each reduction. Within the ThyPRO-39 symptoms of hypothyroidism are assessed as a possible adverse effect. All adverse events reported spontaneously by the participant or their physician are recorded and followed up. It is at the discretion of the GP to decide to either continue the withdrawal of levothyroxine or to maintain or increase the dosage. Serious adverse events are sought out actively and reported to the sponsor, the accredited Medical Ethics Committee (MEC), and competent authority using electronic case report forms.

Monitoring

At regular intervals, the DSMB advises the study project group and sponsor (Leiden University Medical Center [LUMC]) whether it is safe and appropriate to continue the study. Periodic routine reports on the progress of the study are sent to the DSMB for review. Serious adverse events are reported immediately. No interim analyses for efficacy or futility will be performed. The DSMB meets at least 5 times and is composed of medical experts and a biostatistician without any involvement in the study as investigators or as study participant care physicians. The DSMB members are professor J. Wouter Jukema (Chair; Department of Cardiology, LUMC), Dr. Marieke Snel (Department of Internal Medicine – Endocrinology, LUMC), and Dr. Ir. Nan van Geloven (Department of Biomedical Data Sciences, LUMC).

Annual study monitoring visits are conducted by an independent clinical research associate (LUMC) according to a study-specific monitoring plan.

For peer review only - http://bmjopen.bmj.com/site/about/guidelines.xhtml

A summary of the study progress is submitted to the MEC yearly, including the date of inclusion of the first participant, the number of participants included, the number of participants that have completed the trial, serious adverse events, and amendments.

Patient and Public Involvement statement

An online survey (July 2017) revealed that 92% of Dutch GP practices find that investigating the effects of levothyroxine treatment discontinuation is relevant to primary care. The Older People Advisory Board Health and Wellbeing has carefully reviewed the research proposal and their comments were used for improvement. In a letter of recommendation, the Older People Advisory Board Health and Wellbeing has given its full support and endorsement. The results will be discussed with the Older People Advisory Board Health and Wellbeing as well.

For peer review only

For peer review only - http://bmjopen.bmj.com/site/about/guidelines.xhtml

ETHICS AND DISSEMINATION

This study will be conducted according to the principles of the Declaration of Helsinki (amended most recently in October 2013), in accordance with the Medical Research Involving Human Subjects Act (WMO), the Guideline for Good Clinical Practice (May 1996), and in full conformity to any applicable state or local regulations. The protocol has been approved by the accredited MEC (Medisch Ethische Toetsingscommissie Leiden-Den Haag-Delft).

To comply with the general social responsibility associated with clinical research, the study results will be proactively disseminated to the general public and key public health stakeholders including Schildklier Organisatie Nederland, the patient advocacy group. The findings will be submitted for peer-reviewed publication in national and international medical journals.

AUTHORS' CONTRIBUTIONS

Concept and design: RP, RDP, OD, SM, and JG. Drafting of the manuscript: JR, RP, and JG. Critical revision of the manuscript: RDP, OD, and SM. All authors read and approved the final version.

FUNDING STATEMENT

This work was supported by ZonMw, program 'Primary Care and Elderly Care', grant number 839110026. The funder had no role in the design and conduct of the study; collection, management analysis, and interpretation of the data; preparation, review, or approval of the manuscript; or the decision to submit the manuscript for publication.

COMPETING INTERESTS STATEMENT

Ravensberg receives a grant from ZonMw (839110026) during the conduct of the study.

Dr. Du Puy declares no conflict of interest.

Prof. Dr. Mooijaart declares no conflict of interest.

Prof. Dr. Dekkers declares no conflict of interest.

Dr. Poortvliet receives a grant from ZonMw (839110026) during the conduct of the study.

Prof. Dr. Gussekloo receives a grant from ZonMw (839110026) during the conduct of the study.

For peer review only - http://bmjopen.bmj.com/site/about/guidelines.xhtml

REFERENCES

1. ClinCalc. DrugStats 2021 Update - The Most Commonly Prescribed Drugs in the United States https://clincalc.com/blog/2021/09/clincalc-drugstats-2021-update-the-most-commonly-prescribed-drugs-in-the-united-states/?s=levothyroxine2022. Accessed on 4 Aug 2022.
2. Razvi S, Korevaar TIM, Taylor P. Trends, Determinants, and Associations of Treated Hypothyroidism in the United Kingdom, 2005-2014. Thyroid. 2019;29(2):174-82.
3. Jonklaas J, DeSale S. The ages and TSH values of patients being prescribed levothyroxine. Ther Adv Endocrinol Metab. 2020;11:2042018820937896.
4. Statista. Number of levothyroxine prescriptions in the U.S. from 2004-2019. https://www.statista.com/statistics/780284/levothyroxine-prescriptions-number-in-the-us/2022 [updated Feb 3, 2022]. Accessed on 4 Aug 2022.
5. Rodriguez-Gutierrez R, Maraka S, Ospina NS, Montori VM, Brito JP. Levothyroxine overuse: time for an about face? Lancet Diabetes Endocrinol. 2017;5(4):246-8.
6. McDermott MT, Ridgway EC. Subclinical hypothyroidism is mild thyroid failure and should be treated. J Clin Endocrinol Metab. 2001;86(10):4585-90.
7. Brito JP, Ross JS, El Kawkgi OM, Maraka S, Deng Y, Shah ND, et al. Levothyroxine Use in the United States, 2008-2018. JAMA Intern Med. 2021.
8. Taylor PN, Iqbal A, Minassian C, Sayers A, Draman MS, Greenwood R, et al. Falling threshold for treatment of borderline elevated thyrotropin levels-balancing benefits and risks: evidence from a large community-based study. JAMA Intern Med. 2014;174(1):32-9.
9. Medici BB, Nygaard B, la Cour JL, Grand MK, Siersma V, Nicolaisdottir DR, et al. Changes in Prescription Routines for Treating Hypothyroidism Between 2001 and 2015: An Observational Study of 929,684 Primary Care Patients in Copenhagen. Thyroid. 2019;29(7):910-9.
10. Ross DS. Treating hypothyroidism is not always easy: When to treat subclinical hypothyroidism, TSH goals in the elderly, and alternatives to levothyroxine monotherapy. J Intern Med. 2021.
11. Feller M, Snel M, Moutzouri E, Bauer DC, de Montmollin M, Aujesky D, et al. Association of Thyroid Hormone Therapy With Quality of Life and Thyroid-Related Symptoms in Patients With Subclinical Hypothyroidism: A Systematic Review and Meta-analysis. Jama. 2018;320(13):1349-59.
12. Stott DJ, Rodondi N, Kearney PM, Ford I, Westendorp RGJ, Mooijaart SP, et al. Thyroid Hormone Therapy for Older Adults with Subclinical Hypothyroidism. N Engl J Med. 2017;376(26):2534-44.
13. Mooijaart SP, Du Puy RS, Stott DJ, Kearney PM, Rodondi N, Westendorp RGJ, et al. Association Between Levothyroxine Treatment and Thyroid-Related Symptoms Among Adults Aged 80 Years and Older With Subclinical Hypothyroidism. Jama. 2019:1-11.
14. Hall R, Scanlon MF. Hypothyroidism: clinical features and complications. Clin Endocrinol Metab. 1979;8(1):29-38.
15. Rubinoff H, Fireman BH. Testing for recovery of thyroid function after withdrawal of long-term suppression therapy. J Clin Epidemiol. 1989;42(5):417-20.
16. Coll PP, Abourizk NN. Successful withdrawal of thyroid hormone therapy in nursing home patients. J Am Board Fam Pract. 2000;13(6):403-7.
17. Hamblin PS, Dyer SA, Mohr VS, Le Grand BA, Lim CF, Tuxen DV, et al. Relationship between thyrotropin and thyroxine changes during recovery from severe hypothyroxinemia of critical illness. J Clin Endocrinol Metab. 1986;62(4):717-22.
18. Virgini VS, Wijsman LW, Rodondi N, Bauer DC, Kearney PM, Gussekloo J, et al. Subclinical thyroid dysfunction and functional capacity among elderly. Thyroid. 2014;24(2):208-14.
19. Surks MI, Hollowell JG. Age-specific distribution of serum thyrotropin and antithyroid antibodies in the US population: implications for the prevalence of subclinical hypothyroidism. J Clin Endocrinol Metab. 2007;92(12):4575-82.

For peer review only

For peer review only - http://bmjopen.bmj.com/site/about/guidelines.xhtml

20. Canaris GJ, Manowitz NR, Mayor G, Ridgway EC. The Colorado thyroid disease prevalence study. Arch Intern Med. 2000;160(4):526-34.
21. Somwaru LL, Arnold AM, Joshi N, Fried LP, Cappola AR. High frequency of and factors associated with thyroid hormone over-replacement and under-replacement in men and women aged 65 and over. J Clin Endocrinol Metab. 2009;94(4):1342-5.
22. Bauer DC, Ettinger B, Nevitt MC, Stone KL. Risk for fracture in women with low serum levels of thyroid-stimulating hormone. Ann Intern Med. 2001;134(7):561-8.
23. Auer J, Scheibner P, Mische T, Langsteger W, Eber O, Eber B. Subclinical hyperthyroidism as a risk factor for atrial fibrillation. Am Heart J. 2001;142(5):838-42.
24. Mandel SJ, Brent GA, Larsen PR. Levothyroxine therapy in patients with thyroid disease. Ann Intern Med. 1993;119(6):492-502.
25. Burgos N, Toloza FJK, Singh Ospina NM, Brito JP, Salloum RG, Hassett LC, et al. Clinical Outcomes After Discontinuation of Thyroid Hormone Replacement: A Systematic Review and Meta-Analysis. Thyroid. 2021;31(5):740-751.
26. Hernan MA and Robins JM. Causal Inference. 2018, Boca Raton: Chapman & Hall/CRC, forthcoming.
27. Glasziou P, Chalmers I, Rawlins M, McCulloch P. When are randomised trials unnecessary? Picking signal from noise. BMJ. 2007;334(7589):349-51.
28. Blom J, den Elzen W, van Houwelingen AH, Heijmans M, Stijnen T, Van den Hout W, et al. Effectiveness and cost-effectiveness of a proactive, goal-oriented, integrated care model in general practice for older people. A cluster randomised controlled trial: Integrated Systematic Care for older People--the ISCOPE study. Age Ageing. 2016;45(1):30-41.
29. van Houwelingen AH, den Elzen WP, le Cessie S, Blom JW, Gussekloo J. Consequences of interaction of functional, somatic, mental and social problems in community-dwelling older people. PLoS One. 2015;10(4):e0121013.
30. Reeve E, Low LF, Shakib S, Hilmer SN. Development and Validation of the Revised Patients' Attitudes Towards Deprescribing (rPATD) Questionnaire: Versions for Older Adults and Caregivers. Drugs Aging. 2016;33(12):913-28.
31. Atkinson MJ, Kumar R, Cappelleri JC, Hass SL. Hierarchical construct validity of the treatment satisfaction questionnaire for medication (TSQM version II) among outpatient pharmacy consumers. Value Health. 2005;8 Suppl 1:S9-s24.
32. Watt T, Bjorner JB, Groenvold M, Cramon P, Winther KH, Hegedüs L, et al. Development of a Short Version of the Thyroid-Related Patient-Reported Outcome ThyPRO. Thyroid. 2015;25(10):1069-79.
33. Wong CK, Lang BH, Lam CL. A systematic review of quality of thyroid-specific health-related quality-of-life instruments recommends ThyPRO for patients with benign thyroid diseases. J Clin Epidemiol. 2016;78:63-72.
34. EuroQol--a new facility for the measurement of health-related quality of life. Health Policy. 1990;16(3):199-208.
35. Krabbe PF, Stouthard ME, Essink-Bot ML, Bonsel GJ. The effect of adding a cognitive dimension to the EuroQol multiattribute health-status classification system. J Clin Epidemiol. 1999;52(4):293-301.
36. Geraerds A, Bonsel GJ, Janssen MF, Finch AP, Polinder S, Haagsma JA. Methods Used to Identify, Test, and Assess Impact on Preferences of Bolt-Ons: A Systematic Review. Value Health. 2021;24(6):901-16.
37. Brehaut JC, O'Connor AM, Wood TJ, Hack TF, Siminoff L, Gordon E, et al. Validation of a decision regret scale. Med Decis Making. 2003;23(4):281-92.
38. Machin D, Campbell MJ, Tan SB, Tan SH. Sample size tables for clinical studies. 3 ed. 2009, West Sussex: Wiley-Blackwell.
39. Rizzolo PJ, Fischer PM. Re-evaluation of thyroid hormone status after long-term hormone therapy. J Fam Pract. 1982;14(6):1017-21.

For peer review only - http://bmjopen.bmj.com/site/about/guidelines.xhtml

40. Bekkering GE, Agoritsas T, Lytvyn L, Heen AF, Feller M, Moutzouri E, et al. Thyroid hormones treatment for subclinical hypothyroidism: a clinical practice guideline. BMJ. 2019;365:l2006.
41. Sawin CT, Geller A, Hershman JM, Castelli W, Bacharach P. The aging thyroid. The use of thyroid hormone in older persons. Jama. 1989;261(18):2653-5.

For peer review only

Figure 1. Flow of participants

For peer review only

For peer review only - http://bmjopen.bmj.com/site/about/guidelines.xhtml

For peer review only

For peer review only - http://bmjopen.bmj.com/site/about/guidelines.xhtml

Figure 1. Flow of participants

Assessment of eligibility of patients and invitation to participate in the study by GP

↓

Written informed consent

↓

Control phase

- pre-baseline measurements (T-12): thyroid function (exclusion if TSH ≥ 10), descriptives, and secondary outcome measures

↓

Discontinuation phase (T0 – Tx)

1. Baseline measurements (T0): thyroid function and secondary outcomes
2. Consultation by GP
3. Dose-lowering of levothyroxine
4. Control measurement of thyroid function after 6 weeks

5. Repetition of steps 2, 3, and 4 until complete discontinuation of levothyroxine:
   - provided that TSH < 10 mIU/L and FT4 within the reference range

↓

Follow-up phase (Tx-T52)

- Final visit 1 year after the start of the discontinuation phase (T52): thyroid function and secondary outcome measures

For peer review only - http://bmjopen.bmj.com/site/about/guidelines.xhtml

For peer review only

For peer review only - http://bmjopen.bmj.com/site/about/guidelines.xhtml

STANDARD PROTOCOL ITEMS: RECOMMENDATIONS FOR INTERVENTIONAL TRIALS

SPIRIT 2013 Checklist: Recommended items to address in a clinical trial protocol and related documents*

Section/item	Item No	Description	
**Administrative information**			
Title	1	Descriptive title identifying the study design, population, interventions, and, if applicable, trial acronym	page 1
Trial registration	2a	Trial identifier and registry name. If not yet registered, name of intended registry	page 2
	2b	All items from the World Health Organization Trial Registration Data Set	
Protocol version	3	Date and version identifier	page 2
Funding	4	Sources and types of financial, material, and other support	page 14
Roles and responsibilities	5a	Names, affiliations, and roles of protocol contributors	page 1
	5b	Name and contact information for the trial sponsor	
	5c	Role of study sponsor and funders, if any, in study design; collection, management, analysis, and interpretation of data; writing of the report; and the decision to submit the report for publication, including whether they will have ultimate authority over any of these activities	page 14
	5d	Composition, roles, and responsibilities of the coordinating centre, steering committee, endpoint adjudication committee, data management team, and other individuals or groups overseeing the trial, if applicable (see Item 21a for data monitoring committee)	not applicable
**Introduction**			
Background and rationale	6a	Description of research question and justification for undertaking the trial, including summary of relevant studies (published and unpublished) examining benefits and harms for each intervention	page 4
	6b	Explanation for choice of comparators	
Objectives	7	Specific objectives or hypotheses	page 5
Trial design	8	Description of trial design including type of trial (eg, parallel group, crossover, factorial, single group), allocation ratio, and framework (eg, superiority, equivalence, noninferiority, exploratory)	page 5

For peer review only - http://bmjopen.bmj.com/site/about/guidelines.xhtml

Methods: Participants, interventions, and outcomes

Study setting	9	Description of study settings (eg, community clinic, academic hospital) and list of countries where data will be collected. Reference to where list of study sites can be obtained	page 6
Eligibility criteria	10	Inclusion and exclusion criteria for participants. If applicable, eligibility criteria for study centres and individuals who will perform the interventions (eg, surgeons, psychotherapists)	page 6-7
Interventions	11a	Interventions for each group with sufficient detail to allow replication, including how and when they will be administered	page 7-8
	11b	Criteria for discontinuing or modifying allocated interventions for a given trial participant (eg, drug dose change in response to harms, participant request, or improving/worsening disease)	page 8
	11c	Strategies to improve adherence to intervention protocols, and any procedures for monitoring adherence (eg, drug tablet return, laboratory tests)	page 7-8
	11d	Relevant concomitant care and interventions that are permitted or prohibited during the trial	not applicable
Outcomes	12	Primary, secondary, and other outcomes, including the specific measurement variable (eg, systolic blood pressure), analysis metric (eg, change from baseline, final value, time to event), method of aggregation (eg, median, proportion), and time point for each outcome. Explanation of the clinical relevance of chosen efficacy and harm outcomes is strongly recommended	page 8-9, 11
Participant timeline	13	Time schedule of enrolment, interventions (including any run-ins and washouts), assessments, and visits for participants. A schematic diagram is highly recommended (see Figure)	figure 1
Sample size	14	Estimated number of participants needed to achieve study objectives and how it was determined, including clinical and statistical assumptions supporting any sample size calculations	page 11
Recruitment	15	Strategies for achieving adequate participant enrolment to reach target sample size	page 6

Methods: Assignment of interventions (for controlled trials) not applicable

Allocation:

Sequence generation	16a	Method of generating the allocation sequence (eg, computer-generated random numbers), and list of any factors for stratification. To reduce predictability of a random sequence, details of any planned restriction (eg, blocking) should be provided in a separate document that is unavailable to those who enrol participants or assign interventions	

For peer review only - http://bmjopen.bmj.com/site/about/guidelines.xhtml

Allocation concealment mechanism	16b	Mechanism of implementing the allocation sequence (eg, central telephone; sequentially numbered, opaque, sealed envelopes), describing any steps to conceal the sequence until interventions are assigned	
Implementation	16c	Who will generate the allocation sequence, who will enrol participants, and who will assign participants to interventions	
Blinding (masking)	17a	Who will be blinded after assignment to interventions (eg, trial participants, care providers, outcome assessors, data analysts), and how	
	17b	If blinded, circumstances under which unblinding is permissible, and procedure for revealing a participant's allocated intervention during the trial	
**Methods: Data collection, management, and analysis**			
Data collection methods	18a	Plans for assessment and collection of outcome, baseline, and other trial data, including any related processes to promote data quality (eg, duplicate measurements, training of assessors) and a description of study instruments (eg, questionnaires, laboratory tests) along with their reliability and validity, if known. Reference to where data collection forms can be found, if not in the protocol	page 8-9
	18b	Plans to promote participant retention and complete follow-up, including list of any outcome data to be collected for participants who discontinue or deviate from intervention protocols	
Data management	19	Plans for data entry, coding, security, and storage, including any related processes to promote data quality (eg, double data entry; range checks for data values). Reference to where details of data management procedures can be found, if not in the protocol	page 11
Statistical methods	20a	Statistical methods for analysing primary and secondary outcomes. Reference to where other details of the statistical analysis plan can be found, if not in the protocol	page 11-12
	20b	Methods for any additional analyses (eg, subgroup and adjusted analyses)	page 11-12
	20c	Definition of analysis population relating to protocol non-adherence (eg, as randomised analysis), and any statistical methods to handle missing data (eg, multiple imputation)	page 11-12
**Methods: Monitoring**			
Data monitoring	21a	Composition of data monitoring committee (DMC); summary of its role and reporting structure; statement of whether it is independent from the sponsor and competing interests; and reference to where further details about its charter can be found, if not in the protocol. Alternatively, an explanation of why a DMC is not needed	page 12

	21b	Description of any interim analyses and stopping guidelines, including who will have access to these interim results and make the final decision to terminate the trial	
Harms	22	Plans for collecting, assessing, reporting, and managing solicited and spontaneously reported adverse events and other unintended effects of trial interventions or trial conduct	page 12
Auditing	23	Frequency and procedures for auditing trial conduct, if any, and whether the process will be independent from investigators and the sponsor	page 12
**Ethics and dissemination**			
Research ethics approval	24	Plans for seeking research ethics committee/institutional review board (REC/IRB) approval	page 14
Protocol amendments	25	Plans for communicating important protocol modifications (eg, changes to eligibility criteria, outcomes, analyses) to relevant parties (eg, investigators, REC/IRBs, trial participants, trial registries, journals, regulators)	
Consent or assent	26a	Who will obtain informed consent or assent from potential trial participants or authorised surrogates, and how (see Item 32)	page 6
	26b	Additional consent provisions for collection and use of participant data and biological specimens in ancillary studies, if applicable	not applicable
Confidentiality	27	How personal information about potential and enrolled participants will be collected, shared, and maintained in order to protect confidentiality before, during, and after the trial	page 11
Declaration of interests	28	Financial and other competing interests for principal investigators for the overall trial and each study site	page 14
Access to data	29	Statement of who will have access to the final trial dataset, and disclosure of contractual agreements that limit such access for investigators	page 11
Ancillary and post-trial care	30	Provisions, if any, for ancillary and post-trial care, and for compensation to those who suffer harm from trial participation	not applicable
Dissemination policy	31a	Plans for investigators and sponsor to communicate trial results to participants, healthcare professionals, the public, and other relevant groups (eg, via publication, reporting in results databases, or other data sharing arrangements), including any publication restrictions	page 14
	31b	Authorship eligibility guidelines and any intended use of professional writers	
	31c	Plans, if any, for granting public access to the full protocol, participant-level dataset, and statistical code	

For peer review only - http://bmjopen.bmj.com/site/about/guidelines.xhtml

Appendices

Informed consent materials	32	Model consent form and other related documentation given to participants and authorised surrogates
Biological specimens	33	Plans for collection, laboratory evaluation, and storage of biological specimens for genetic or molecular analysis in the current trial and for future use in ancillary studies, if applicable

*It is strongly recommended that this checklist be read in conjunction with the SPIRIT 2013 Explanation & Elaboration for important clarification on the items. Amendments to the protocol should be tracked and dated. The SPIRIT checklist is copyrighted by the SPIRIT Group under the Creative Commons "Attribution-NonCommercial-NoDerivs 3.0 Unported" license.

For peer review only

BMJ Open

The effects of discontinuation of levothyroxine treatment in older adults: Protocol for a self-controlled trial

Journal:	*BMJ Open*
Manuscript ID	bmjopen-2022-070741.R1
Article Type:	Protocol
Date Submitted by the Author:	17-Mar-2023
Complete List of Authors:	Ravensberg, A.; Leiden University Medical Center, Department of Public Health and Primary Care<br>Poortvliet, Rosalinde K. E.; Leiden University Medical Center, Department of Public Health and Primary Care; Leiden University Medical Center, University Network for the Care sector Zuid-Holland<br>Du Puy, Robert ; Leiden University Medical Center, Department of Public Health and Primary Care<br>Dekkers, Olaf; Leiden University Medical Center, Endocrinology and Metabolic Disorders<br>Mooijaart, Simon; Leiden University Medical Center, Department of Gerontology and Geriatrics<br>Gussekloo, Jacobijn; Leiden University Medical Center, Department of Public Health and Primary Care; Leiden University Medical Center, Department of Gerontology and Geriatrics
**Primary Subject Heading**:	Diabetes and endocrinology
Secondary Subject Heading:	General practice / Family practice, Geriatric medicine
Keywords:	Thyroid disease < DIABETES & ENDOCRINOLOGY, Quality of Life, GENERAL MEDICINE (see Internal Medicine), PRIMARY CARE, GERIATRIC MEDICINE, Aged

SCHOLARONE™
Manuscripts

TITLE

The effects of discontinuation of levothyroxine treatment in older adults: Protocol for a self-controlled trial

Authors

A. Janneke Ravensberg[1]

R.K.E. Poortvliet[1, 2]

R.S. Du Puy[1]

O.M. Dekkers[3]

S.P. Mooijaart[4]

J.G. Gussekloo[1]

[1]Department of Public Health and Primary Care, Leiden University Medical Center, Leiden, the Netherlands.

[2] University Network for the Care sector Zuid-Holland, Leiden University Medical Center, Leiden, the Netherlands

[3]Department of Endocrinology and Metabolic Disorders, Leiden University Medical Center, Leiden, the Netherlands.

[4]Department of Gerontology and Geriatrics, Leiden University Medical Center, Leiden, the Netherlands.

Corresponding author:

Janneke Ravensberg

Leiden University Medical Center, Department of Public Health and Primary Care

Postzone V-0-P; PO BOX 9600; 2300 RC Leiden, the Netherlands

a.j.j.ravensberg@lumc.nl

tel: +31 71 526 8444; fax: +31 71 526 8259

Wordcount: 3445

ABSTRACT

Background Many older persons use the thyroid hormone levothyroxine which is often continued for life. Scientifically, there is much uncertainty whether simple continuation is the optimal approach. First, the physical need for levothyroxine can decrease with age thereby posing a higher risk of overtreatment and adverse effects. Second, large trials in subclinical hypothyroidism have shown no benefit for the use of levothyroxine. Interestingly, guidelines do not address re-evaluation of the indication. This self-controlled trial aims to determine the effects of discontinuation of levothyroxine treatment in older adults.

Methods and analysis Participants are community-dwelling subjects aged ≥ 60 years using levothyroxine continuously at a stable dosage of ≤ 150 microgram and a level of TSH <10 mU/L. After a control period of 12 weeks, levothyroxine treatment is discontinued gradually using a step-wise approach with regular monitoring of thyroid function guided by their general practitioner. The primary outcome is the proportion of participants withdrawn from levothyroxine while maintaining a free T4 level within the reference range and a TSH level <10 mU/L, 52 weeks after the start of discontinuation. Secondary outcomes are compared to the control period (self-controlled) and include amongst others, the effects on thyroid-specific and general health-related quality of life. Furthermore, patients' attitudes towards deprescribing and regret regarding discontinuing levothyroxine treatment will be recorded. A total of 518 participants will be recruited to estimate the expected proportion of 50% with a 95% confidence interval ranging from 45% to 55%.

Ethics and dissemination Approval was obtained from the institutional medical ethics committee. The Older People Advisory Board Health and Wellbeing has reviewed the research proposal and their comments were used for improvement. In line with the funding policies of the grant organisation funding this study, the study results will be proactively disseminated to the general public and key public health stakeholders.

Trial registration Netherlands Trial Register (NTR); NL7978; registered 22 August 2019. The NTR is no longer available; re-registration with ClinicalTrials.gov is pending.

Protocol version version 6.0; 20 September 2022.

For peer review only

For peer review only - http://bmjopen.bmj.com/site/about/guidelines.xhtml

Keywords (MeSH descriptors)

1. Humans
2. Aged;
3. Aged, 80 and over;
4. Thyroxine;
5. Deprescriptions;
6. Observational Study;
7. Thyroid Diseases;
8. Hypothyroidism;
9. Quality of Life;
10. Health Status;
11. Personal Satisfaction;
12. General Practitioners

Strengths and limitations of this study

- This is a self-controlled trial to assess the effect of discontinuation of levothyroxine treatment specifically in older adults (aged ≥ 60 years), using both thyroid function and thyroid-related quality of life as outcome parameters.
- Stepwise dose reduction with regular measurements of thyroid function reduces the risk of prolonged periods of undertreatment potentially resulting in symptoms of hypothyroidism.
- The primary endpoint, successful withdrawal of levothyroxine, is a biochemically defined endpoint, which is not affected by the unblinded design of the study.
- The unblinded design of this study may introduce performance bias and detection bias in the outcomes of the secondary end points.

For peer review only

For peer review only - http://bmjopen.bmj.com/site/about/guidelines.xhtml

INTRODUCTION

Levothyroxine is one of the most widely prescribed drugs and is currently ranked in the top 3 of total prescriptions in the United States (US) and United Kingdom (UK) (1, 2). It is most frequently prescribed and initiated in older adults (aged 50-70 years) (3). Notably, levothyroxine prescriptions have increased substantially, from 2.3% to 3.5% over the past decades despite a low prevalence (0.1-1.9%) and a stable incidence of overt hypothyroidism (2, 4). A contributing factor to the rise in levothyroxine prescriptions is likely an increase in the treatment of subclinical hypothyroidism (5). Subclinical hypothyroidism is a common condition defined by an elevated level of thyroid-stimulating hormone (TSH) and a free T4 (fT4) level within the normal reference range. The prevalence of subclinical hypothyroidism increases with age (5-20% in men and women older than 60 years) (6). A recent US study showed that most individuals who started on levothyroxine were diagnosed with subclinical hypothyroidism (65.7%) (7). Furthermore, studies indicate that TSH levels at the initiation of treatment are falling (8, 9), which is worrisome because it may increase the chance of ineffective treatment (10). This holds especially for older adults with subclinical hypothyroidism with TSH levels <10 mU/L since recent randomized clinical trials show that levothyroxine treatment does not improve thyroid-related symptoms, quality of life, depressive symptoms, or cognitive function in older people (11-13).

Treatment with levothyroxine for both overt and subclinical hypothyroidism, is often continued for life, as current guidelines do not advise re-evaluation of the indication and effect (8, 14). Initial indications for treatment are not always well documented and can be inaccurate or even inappropriate (15). Patients may have started levothyroxine in the past for a diagnosis made during transient hypothyroidism (15-17). Furthermore, thyroid (dys-)function varies over time (18), and thyroid metabolism changes with age (19). Therefore, it is likely that a considerable proportion of older adults are treated with levothyroxine without a clear indication according to current guidelines. This could be harmful, considering that oversuppression (TSH < 0.45 mU/L) has been reported in 41% of older levothyroxine users (, 20) and increases the risk of bone fractures and atrial fibrillation (21, 22). The risk of oversuppression increases with treatment duration (8) and although guidelines warrant careful medication monitoring, levothyroxine dosages may remain unchanged for a long time (23).

Taking into account the high levothyroxine use in older age, the controversy regarding treatment of subclinical hypothyroidism, the lack of re-evaluation of indications, and the health risks associated with oversuppression, it can be hypothesized that discontinuation of levothyroxine treatment can be achieved without negative consequences for a considerable proportion of older adults. A meta-analysis based on observational studies including participants aged 2-81 years suggested that approximately 37% remain euthyroid after thyroid hormone discontinuation (24).

For peer review only - http://bmjopen.bmj.com/site/about/guidelines.xhtml

However, the evidence was of low quality, the adult participants were mostly middle-aged, and no study followed a systematic approach for deprescribing levothyroxine.

Therefore, the primary aim of this self-controlled trial is to investigate what proportion of community-dwelling levothyroxine users aged 60 years and older can withdraw from levothyroxine treatment successfully. Secondary aims are: 1. to study what proportion of participants can achieve a substantial dose reduction of levothyroxine at 52 weeks a) defined as ≥50% and b) defined by the participants themselves; 2. to study the effect of discontinuation of levothyroxine treatment at 52 weeks on a) thyroid-related quality of life and b) general health; 3. to determine how participants reflect on their decision to discontinue levothyroxine treatment.

For peer review only

METHODS

Study design

This is a self-controlled trial investigating the stepwise discontinuation of levothyroxine treatment in adults aged 60 years and older with a follow-up of 1 year. Outcomes will be measured 12 weeks before the start of discontinuation, at the start of discontinuation, at 6 weeks, at the end of discontinuation, and after 52 weeks. The outcomes will be compared within participants. In this way, the research questions can be studied validly in a self-controlled design, without the need for an extra control group. The formal role of a control group in a randomized trial is to estimate what would have happened in the experimental arm in the absence of the intervention (25). This effect is immediately clear in our study: levothyroxine treatment is simply continued in almost all patients. Therefore, a control group in our study will only reveal information that is already known (26). The primary outcome of the study is based on a biochemically defined thyroid function status, an fT4 level within the reference range and a TSH level <10 mU/L. Since this biochemically defined outcome is independent of participants' views or experiences, is not influenced by personal interpretation, and is therefore not affected by an unblinded design, we considered a control group as not required. A TSH level of <10 mU/L was based on the results from the recently published randomized clinical trials on levothyroxine treatment in older adults with subclinical hypothyroidism (11-13).

Study setting

Community-dwelling participants are recruited from general practitioner (GP) practices in the Netherlands. Participating GPs identify eligible patients for whom they are the primary care giver concerning levothyroxine treatment, from electronic medical records using the in- and exclusion criteria mentioned below. GPs may exclude patients when they feel study participation could harm their patient. Reasons for exclusion are collected. Selected patients receive an invitation to participate in the study and an information leaflet from their GP (see Appendix 1). Written informed consent is obtained from all patients who are willing to participate in the study (see Appendix 1). Participants are enrolled by the study centre.

Study population

A participant must meet all of the following inclusion criteria:

1. Age ≥ 60 years;
2. Using any levothyroxine mono-therapy medicament (ATC: H03AA01) at a stable dosage for ≥ 1 year.

For peer review only - http://bmjopen.bmj.com/site/about/guidelines.xhtml

Exclusion criteria are:

1. Last measurement of TSH ≥10 mU/L during levothyroxine treatment;
2. The dose of treatment is > 150 mcg levothyroxine per day (for safety reasons);
3. The current reason for levothyroxine treatment: patients with a history of thyroidectomy; radioactive iodine treatment or neck irradiation; congenital hypothyroidism; secondary hypothyroidism, or concurrent amiodarone or lithium use;
4. Concurrent treatment with liothyronine (the synthetic form of thyroid hormone T3), thiamazole, carbimazole, or propylthiouracil (inhibitors of T3 and T4 synthesis);
5. Diagnosis of heart failure NYHA grade IV;
6. Participation in ongoing trials of therapeutic interventions;
7. Life-expectancy < 6 months;
8. Diagnosis of dementia;
9. Incapacitated adults;
10. Persons planning to move out of the region in which the study is being conducted in the next months.

Study phases and interventions

This study consists of 3 phases: a control phase of 12 weeks (T-12 to T0), the discontinuation phase (minimum of 6 weeks, maximum of 36 weeks; T0 to Tx), and a follow-up phase (from the end of discontinuation till 52 weeks after the start of discontinuation; Tx to T52) (see Figure 1).

Control phase Participants enter the study during a control period with pre-baseline measurements of thyroid function and secondary outcome parameters to determine natural variation after which treatment with levothyroxine is continued per usual care for at least 12 weeks (see Table 1). Participants with a pre-baseline TSH level ≥10 mU/L do not proceed to the discontinuation phase (exclusion). When a participant presents with a suppressed TSH (indicative of oversuppletion with levothyroxine), the discontinuation phase can be started immediately. Participants are requested to complete their questionnaires prior to thyroid function assessment so the results do not influence their answers. Furthermore, they are instructed to schedule all laboratory visits at the same time preferably in the morning before ingesting levothyroxine and consumption of food and/or drinks.

Discontinuation phase The discontinuation phase starts with baseline measurements of thyroid function and secondary outcome parameters (see Table 1), followed by a consultation of the participant with their GP during which discontinuation of levothyroxine is initiated, except when TSH level ≥10 mU/L. Levothyroxine is discontinued stepwise, and at intervals of ≥ 6 weeks to ascertain a steady-state concentration has been reached before the following control visit. This is in line with

For peer review only - http://bmjopen.bmj.com/site/about/guidelines.xhtml

standard clinical practice. During each '6-week' visit, the GP evaluates thyroid function and symptoms with the participant. Upon agreement, the daily dose of levothyroxine can then be lowered provided thyroid function is within the predefined limits, i.e. a normal fT4 and TSH < 10 mU/L. With each step, the dose of levothyroxine is reduced by approximately 25 mcg (the use of whole tablets is preferred) until a daily dose of 25 mcg or less is reached. The GP is free to adjust the size of the dose reduction if necessary. The maximum dose reduction in medication is 50 mcg in the first step. In the final step, the medication is discontinued completely if the laboratory results are still within the predefined limits. The discontinuation phase ends after the evaluation of complete withdrawal of the levothyroxine treatment (i.e. 6 weeks after the final discontinuation step). Secondary outcome parameters are collected at the first '6-week' visit and the end of discontinuation (see Table 1).

Participants are requested to stop the discontinuation phase when a TSH level ≥ 10 mU/L or an fT4 level below the normal range is identified during a control visit and is confirmed by an extra thyroid function measurement within two weeks. When the extra measurement does not confirm a TSH level ≥ 10 mU/L or a low fT4 level, the participant is allowed to proceed with the discontinuation phase. Other factors than thyroid function, such as symptoms, may however be a reason to prematurely stop the discontinuation or restart levothyroxine. Upon stopping the discontinuation phase, it is at the discretion of the physician and the participant to decide whether the dose of levothyroxine at that moment will be maintained or increased. Participants who have stopped the discontinuation phase prematurely will be followed up in accordance with the protocol if possible.

Follow-up phase The follow-up phase ends with a final visit 52 weeks after the start of the discontinuation phase and includes the assessment of thyroid function, current dose of levothyroxine, and secondary outcome parameters (see Table 1).

Adherence Participants are contacted by the research nurse by telephone and/or by mail or email prior to each measurement and in case of missing data, to ensure data quality and to promote participant retention and complete follow-up.

Descriptive study data and outcome measures

Descriptive data recorded at pre-baseline include (see Appendix 2):

1. Age and sex.
2. Lifestyle: smoking, alcohol intake.
3. Medical history.
4. Complex health problems (Integrated Systematic Care for Older Persons [ISCOPE] questionnaire) (27, 28).

For peer review only - http://bmjopen.bmj.com/site/about/guidelines.xhtml

5. The participant's attitude towards deprescribing (revised Patients' Attitudes Towards Deprescribing [rPATD] questionnaire) (29).
6. Treatment satisfaction for levothyroxine treatment (Treatment Satisfaction Questionnaire for Medication [TSQM]) (30).
7. The participant's view on what is a substantial dose reduction of levothyroxine (Goal Attainment Score [GAS]).

The primary outcome is the proportion of participants that withdraw their thyroid medication successfully (defined as normal fT4 levels and TSH levels <10 mU/L) at 52 weeks after the start of the discontinuation (final follow-up).

Secondary outcome measures determined at baseline and/or final follow-up include (see Appendix 2):

- Thyroid-related quality of life (ThyPRO-39) (31, 32).
- General health-related quality of life (EuroQol-5D, including the EuroQol visual analogue scale (33). Cognitive function is determined as an additional dimension (EuroQol-5D+C) (34, 35).
- Participant's view on whether they achieved a substantial dose reduction of levothyroxine (Goal Attainment Scale [GAS]).
- Participant's view on regret after the decision to withdraw levothyroxine (Decision Regret Scale [DRS]) (36).

To further explore the quality of life during the discontinuation phase, ThyPRO-39 and EuroQol measurements were also determined 6 weeks after the first step of lowering the dose of levothyroxine (T6) and 6 weeks after the last step of dose-lowering (see Table 1).

For peer review only

Table 1. Schedule of measurements

	Study phase					
	**Control phase**	**Discontinuation phase**				**Follow-up phase**
		**Start of step 1**	**Control of step 1**	**Control of steps 2-5**	**Control of last step[c]**	
**Time (weeks)**	**-12**	**0**	**6**	**12, 18, 24, 30**	**6/12/18/24/30/36**	**52**
**Primary Outcome**						
TSH, FT4	X	X	X	X[b]	X	X
**Secondary Outcomes**						
ThyPRO-39	X	X	X		X	X
TSQM	X	X				
EQ5D VAS	X	X	X		X	X
DRS						X
GAS[a]						X
**Descriptives**						
Demographics & clinical characteristics	X					
ISCOPE	X					X
rPATD	X	X				
GAS[a]	X					

Abbreviation: TSH, Thyroid Stimulating Hormone; FT4, Free T4; ThyPRO, Thyroid-Related Quality of Life Patient-Reported Outcome; TSQM, Treatment Satisfaction Questionnaire for Medication; EQ5D, EuroQol 5 Dimensions; VAS, Visual Analogue Scale; DRS, Decision Regret Scale; GAS, Goal Attainment Scale; ISCOPE, Integrated Systematic Care for Older Persons; rPATD, revised Patients' Attitudes Towards Deprescribing.

[a] Substantial dose reduction of levothyroxine; [b] If applicable; [c] End of the discontinuation phase.

For peer review only

Sample size calculation

The sample size calculation is based on the primary outcome, the proportion of participants successfully stopping levothyroxine treatment between baseline and final follow-up at 52 weeks. Since population proportions of successful levothyroxine treatment cessation are unknown (the aim of this study), we estimated a proportion of 50% of the participants that successfully discontinue levothyroxine treatment. An estimated proportion of 50% results in the most conservative estimate (resulting in the highest calculated sample size) and is in line with the limited available evidence (37-39). We have chosen a sample size of 385 participants because this allows us to estimate the expected proportion of 50% with a 95% confidence interval of which the lower and upper limit is expected to deviate by 5 percentage points from the found percentage (i.e. 45% to 55% when the found percentage is indeed 50%).

Taking a maximum of 25% loss to follow up into account over the duration of 15 months (exclusion at T-12 when TSH level ≥ 10 mU/L and/or fT4 below reference range, withdrawal, moving away, or death between T-12 and T52) a maximum of 513 participants will be recruited. Based on a data query from the academic network of general practices in the region of Leiden, it was estimated that the projected sample size will be reached after inclusion of 53 standard general practices.

Data analysis plan

GPs transfer participants' data after each visit using a validated electronic data capture (EDC) system (Castor EDC). Questionnaires are completed on paper or electronically (Castor EDC). All participant data are coded. Only the principal investigators and the research team have access to the key to these codes. The handling of personal data complies with the European General Data Protection Regulation. Database validation checks are run routinely and are tracked and escalated as appropriate.

To determine the proportion of the population that can be withdrawn from levothyroxine treatment, categorical data involving measurements at baseline and final follow-up will be analyzed. Univariable and multivariable logistic regression analyses will be performed to assess factors that are associated with successful discontinuation of levothyroxine treatment. To estimate the effect of discontinuation on secondary outcome parameters measured at baseline and/or final follow-up, descriptive, univariable and multivariable analyses will be performed for the overall group and for the group of participants who successfully discontinued levothyroxine. Measurements pre and post discontinuation will be compared. Variations in thyroid function, thyroid-related quality of life, treatment satisfaction for levothyroxine, general health, and participant's attitude toward deprescribing will be determined using pre-baseline and baseline measurements. All analyses will be carried out with the population of participants who enrolled at baseline (T0). Participants that experience intercurrent events not related to the study procedure resulting in missing data at final follow-up will be excluded. Primary

For peer review only - http://bmjopen.bmj.com/site/about/guidelines.xhtml

and secondary analyses will be repeated with all participants who enrolled at baseline who have data for the outcome variable of interest and have had their final follow-up visit. Statistical significance for the primary outcome requires $P \leq 0.05$.

Safety and Monitoring

Withdrawal

Participants can withdraw their consent for involvement in the study at any time, without an obligation to justify the decision, and without consequences for their usual care. The research team can advise withdrawing participants from the intervention if it is deemed to be in the best interest of the participant. Participants who withdraw from the study are not replaced. The Data Safety and Monitoring Board (DSMB) can advise to stop the study.

(Serious) adverse events

To avoid prolonged periods of thyroid hormone underreplacement potentially resulting in symptoms of hypothyroidism, levothyroxine treatment is discontinued gradually while monitoring thyroid function after each reduction. Within the ThyPRO-39 symptoms of hypothyroidism are assessed as a possible adverse effect. All adverse events (including cardiovascular events) reported spontaneously by the participant or their GP are recorded and followed up. If complaints are reported during the discontinuation phase, these will be assessed by the research nurse. It is at the discretion of the GP to decide to either continue the withdrawal of levothyroxine or to maintain or increase the dosage. Serious adverse events are sought out actively and reported to the sponsor, the accredited Medical Ethics Committee (MEC), and competent authority using electronic case report forms.

Monitoring

At regular intervals, the DSMB advises the study project group and sponsor (Leiden University Medical Center [LUMC]) whether it is safe and appropriate to continue the study. Periodic routine reports on the progress of the study are sent to the DSMB for review. Serious adverse events are reported immediately. No interim analyses for efficacy or futility will be performed. The DSMB meets at least 5 times and is composed of medical experts and a biostatistician without any involvement in the study as investigators or as study participant care physicians. The DSMB members are professor J. Wouter Jukema (Chair; Department of Cardiology, LUMC), Dr. Marieke Snel (Department of Internal Medicine – Endocrinology, LUMC), and Dr. Ir. Nan van Geloven (Department of Biomedical Data Sciences, LUMC).

Annual study monitoring visits are conducted by an independent clinical research associate (LUMC) according to a study-specific monitoring plan.

For peer review only - http://bmjopen.bmj.com/site/about/guidelines.xhtml

A summary of the study progress is submitted to the MEC yearly, including the date of inclusion of the first participant, the number of participants included, the number of participants that have completed the trial, serious adverse events, and amendments.

Roles and responsibilities (committees) are also described in Appendix 3, as is additional trial information regarding trial registration data and trial sponsor.

Patient and Public Involvement statement

An online survey (July 2017) revealed that 92% of Dutch GP practices find that investigating the effects of levothyroxine treatment discontinuation is relevant to primary care. The Older People Advisory Board Health and Wellbeing has carefully reviewed the research proposal and their comments were used for improvement. In a letter of recommendation, the Older People Advisory Board Health and Wellbeing has given its full support and endorsement. The results will be discussed with the Older People Advisory Board Health and Wellbeing as well.

For peer review only - http://bmjopen.bmj.com/site/about/guidelines.xhtml

ETHICS AND DISSEMINATION

This study will be conducted according to the principles of the Declaration of Helsinki (amended most recently in October 2013), in accordance with the Dutch Medical Research Involving Human Subjects Act (WMO), the Guideline for Good Clinical Practice (May 1996), and in full conformity to any applicable state or local regulations. In accordance with Dutch legal requirements (WMO), participants will be insured by an insurance of the LUMC to provide cover for damage to research participants through injury or death caused by the study (see Appendix 1). The protocol has been approved by the accredited MEC (Medisch Ethische Toetsingscommissie Leiden-Den Haag-Delft). Any protocol modifications will be communicated to all relevant parties (including the accredited MEC, the DSMB, participating GPs, participants and trial register).

To comply with the general social responsibility associated with clinical research, the study results will be proactively disseminated to the general public and key public health stakeholders including Schildklier Organisatie Nederland, the patient advocacy group. The findings will be submitted for peer-reviewed publication in national and international medical journals. Publications will be approved by the principle investigators. Individual deidentified participant data can be shared upon request only, when the participant has given permission (i.e. informed consent) for data sharing concerning future research regarding thyroid disease and treatment with levothyroxine. The study protocol and the statistical analysis plan (SAP) will be made available online before publication of the main results from this study.

AUTHORS' CONTRIBUTIONS

Concept and design: JR, RP, RDP, OD, SM, and JG. Drafting of the manuscript: JR, RP, and JG. Critical revision of the manuscript: RDP, OD, and SM. All authors read and approved the final version. All authors agree to be accountable for all aspects of the work in ensuring that questions related to the accuracy or integrity of any part of the work are appropriately investigated and resolved.

FUNDING STATEMENT

This work was supported by ZonMw, program 'Primary Care and Elderly Care', grant number 839110026. The funder had no role in the design and conduct of the study; collection, management analysis, and interpretation of the data; preparation, review, or approval of the manuscript; or the decision to submit the manuscript for publication.

COMPETING INTERESTS STATEMENT

For peer review only - http://bmjopen.bmj.com/site/about/guidelines.xhtml

Ravensberg receives a grant from ZonMw (839110026) during the conduct of the study.

Dr. Du Puy declares no conflict of interest.

Prof. Dr. Mooijaart declares no conflict of interest.

Prof. Dr. Dekkers declares no conflict of interest.

Dr. Poortvliet receives a grant from ZonMw (839110026) during the conduct of the study.

Prof. Dr. Gussekloo receives a grant from ZonMw (839110026) during the conduct of the study.

For peer review only

For peer review only - http://bmjopen.bmj.com/site/about/guidelines.xhtml

REFERENCES

1. ClinCalc. DrugStats 2021 Update - The Most Commonly Prescribed Drugs in the United States https://clincalc.com/blog/2021/09/clincalc-drugstats-2021-update-the-most-commonly-prescribed-drugs-in-the-united-states/?s=levothyroxine2022. Accessed on 4 Aug 2022.
2. Razvi S, Korevaar TIM, Taylor P. Trends, Determinants, and Associations of Treated Hypothyroidism in the United Kingdom, 2005-2014. Thyroid. 2019;29(2):174-82.
3. Jonklaas J, DeSale S. The ages and TSH values of patients being prescribed levothyroxine. Ther Adv Endocrinol Metab. 2020;11:2042018820937896.
4. Statista. Number of levothyroxine prescriptions in the U.S. from 2004-2019. https://www.statista.com/statistics/780284/levothyroxine-prescriptions-number-in-the-us/2022 [updated Feb 3, 2022]. Accessed on 4 Aug 2022.
5. Rodriguez-Gutierrez R, Maraka S, Ospina NS, Montori VM, Brito JP. Levothyroxine overuse: time for an about face? Lancet Diabetes Endocrinol. 2017;5(4):246-8.
6. McDermott MT, Ridgway EC. Subclinical hypothyroidism is mild thyroid failure and should be treated. J Clin Endocrinol Metab. 2001;86(10):4585-90.
7. Brito JP, Ross JS, El Kawkgi OM, Maraka S, Deng Y, Shah ND, et al. Levothyroxine Use in the United States, 2008-2018. JAMA Intern Med. 2021.
8. Taylor PN, Iqbal A, Minassian C, Sayers A, Draman MS, Greenwood R, et al. Falling threshold for treatment of borderline elevated thyrotropin levels-balancing benefits and risks: evidence from a large community-based study. JAMA Intern Med. 2014;174(1):32-9.
9. Medici BB, Nygaard B, la Cour JL, Grand MK, Siersma V, Nicolaisdottir DR, et al. Changes in Prescription Routines for Treating Hypothyroidism Between 2001 and 2015: An Observational Study of 929,684 Primary Care Patients in Copenhagen. Thyroid. 2019;29(7):910-9.
10. Ross DS. Treating hypothyroidism is not always easy: When to treat subclinical hypothyroidism, TSH goals in the elderly, and alternatives to levothyroxine monotherapy. J Intern Med. 2021.
11. Feller M, Snel M, Moutzouri E, Bauer DC, de Montmollin M, Aujesky D, et al. Association of Thyroid Hormone Therapy With Quality of Life and Thyroid-Related Symptoms in Patients With Subclinical Hypothyroidism: A Systematic Review and Meta-analysis. Jama. 2018;320(13):1349-59.
12. Stott DJ, Rodondi N, Kearney PM, Ford I, Westendorp RGJ, Mooijaart SP, et al. Thyroid Hormone Therapy for Older Adults with Subclinical Hypothyroidism. N Engl J Med. 2017;376(26):2534-44.
13. Mooijaart SP, Du Puy RS, Stott DJ, Kearney PM, Rodondi N, Westendorp RGJ, et al. Association Between Levothyroxine Treatment and Thyroid-Related Symptoms Among Adults Aged 80 Years and Older With Subclinical Hypothyroidism. Jama. 2019:1-11.
14. Hall R, Scanlon MF. Hypothyroidism: clinical features and complications. Clin Endocrinol Metab. 1979;8(1):29-38.
15. Rubinoff H, Fireman BH. Testing for recovery of thyroid function after withdrawal of long-term suppression therapy. J Clin Epidemiol. 1989;42(5):417-20.
16. Coll PP, Abourizk NN. Successful withdrawal of thyroid hormone therapy in nursing home patients. J Am Board Fam Pract. 2000;13(6):403-7.
17. Hamblin PS, Dyer SA, Mohr VS, Le Grand BA, Lim CF, Tuxen DV, et al. Relationship between thyrotropin and thyroxine changes during recovery from severe hypothyroxinemia of critical illness. J Clin Endocrinol Metab. 1986;62(4):717-22.
18. Virgini VS, Wijsman LW, Rodondi N, Bauer DC, Kearney PM, Gussekloo J, et al. Subclinical thyroid dysfunction and functional capacity among elderly. Thyroid. 2014;24(2):208-14.
19. Surks MI, Hollowell JG. Age-specific distribution of serum thyrotropin and antithyroid antibodies in the US population: implications for the prevalence of subclinical hypothyroidism. J Clin Endocrinol Metab. 2007;92(12):4575-82.
20. Somwaru LL, Arnold AM, Joshi N, Fried LP, Cappola AR. High frequency of and factors associated with thyroid hormone over-replacement and under-replacement in men and women aged 65 and over. J Clin Endocrinol Metab. 2009;94(4):1342-5.

For peer review only - http://bmjopen.bmj.com/site/about/guidelines.xhtml

21. Bauer DC, Ettinger B, Nevitt MC, Stone KL. Risk for fracture in women with low serum levels of thyroid-stimulating hormone. Ann Intern Med. 2001;134(7):561-8.
22. Auer J, Scheibner P, Mische T, Langsteger W, Eber O, Eber B. Subclinical hyperthyroidism as a risk factor for atrial fibrillation. Am Heart J. 2001;142(5):838-42.
23. Mandel SJ, Brent GA, Larsen PR. Levothyroxine therapy in patients with thyroid disease. Ann Intern Med. 1993;119(6):492-502.
24. Burgos N, Toloza FJK, Singh Ospina NM, Brito JP, Salloum RG, Hassett LC, et al. Clinical Outcomes After Discontinuation of Thyroid Hormone Replacement: A Systematic Review and Meta-Analysis. Thyroid. 2021;31(5):740-751.
25. Hernan MA and Robins JM. Causal Inference. 2018, Boca Raton: Chapman & Hall/CRC, forthcoming.
26. Glasziou P, Chalmers I, Rawlins M, McCulloch P. When are randomised trials unnecessary? Picking signal from noise. BMJ. 2007;334(7589):349-51.
27. Blom J, den Elzen W, van Houwelingen AH, Heijmans M, Stijnen T, Van den Hout W, et al. Effectiveness and cost-effectiveness of a proactive, goal-oriented, integrated care model in general practice for older people. A cluster randomised controlled trial: Integrated Systematic Care for older People--the ISCOPE study. Age Ageing. 2016;45(1):30-41.
28. van Houwelingen AH, den Elzen WP, le Cessie S, Blom JW, Gussekloo J. Consequences of interaction of functional, somatic, mental and social problems in community-dwelling older people. PLoS One. 2015;10(4):e0121013.
29. Reeve E, Low LF, Shakib S, Hilmer SN. Development and Validation of the Revised Patients' Attitudes Towards Deprescribing (rPATD) Questionnaire: Versions for Older Adults and Caregivers. Drugs Aging. 2016;33(12):913-28.
30. Atkinson MJ, Kumar R, Cappelleri JC, Hass SL. Hierarchical construct validity of the treatment satisfaction questionnaire for medication (TSQM version II) among outpatient pharmacy consumers. Value Health. 2005;8 Suppl 1:S9-s24.
31. Watt T, Bjorner JB, Groenvold M, Cramon P, Winther KH, Hegedüs L, et al. Development of a Short Version of the Thyroid-Related Patient-Reported Outcome ThyPRO. Thyroid. 2015;25(10):1069-79.
32. Wong CK, Lang BH, Lam CL. A systematic review of quality of thyroid-specific health-related quality-of-life instruments recommends ThyPRO for patients with benign thyroid diseases. J Clin Epidemiol. 2016;78:63-72.
33. EuroQol--a new facility for the measurement of health-related quality of life. Health Policy. 1990;16(3):199-208.
34. Krabbe PF, Stouthard ME, Essink-Bot ML, Bonsel GJ. The effect of adding a cognitive dimension to the EuroQol multiattribute health-status classification system. J Clin Epidemiol. 1999;52(4):293-301.
35. Geraerds A, Bonsel GJ, Janssen MF, Finch AP, Polinder S, Haagsma JA. Methods Used to Identify, Test, and Assess Impact on Preferences of Bolt-Ons: A Systematic Review. Value Health. 2021;24(6):901-16.
36. Brehaut JC, O'Connor AM, Wood TJ, Hack TF, Siminoff L, Gordon E, et al. Validation of a decision regret scale. Med Decis Making. 2003;23(4):281-92.

37. Rizzolo PJ, Fischer PM. Re-evaluation of thyroid hormone status after long-term hormone therapy. J Fam Pract. 1982;14(6):1017-21.
38. Bekkering GE, Agoritsas T, Lytvyn L, Heen AF, Feller M, Moutzouri E, et al. Thyroid hormones treatment for subclinical hypothyroidism: a clinical practice guideline. BMJ. 2019;365:l2006.
39. Sawin CT, Geller A, Hershman JM, Castelli W, Bacharach P. The aging thyroid. The use of thyroid hormone in older persons. Jama. 1989;261(18):2653-5.

For peer review only - http://bmjopen.bmj.com/site/about/guidelines.xhtml

Figure 1. Flow of participants

For peer review only

For peer review only - http://bmjopen.bmj.com/site/about/guidelines.xhtml

Figure 1. Flow of participants

Assessment of eligibility of patients and invitation to participate in the study by GP

↓

Written informed consent

↓

Control phase

- pre-baseline measurements (T-12): thyroid function (exclusion if TSH ≥ 10), descriptives, and secondary outcome measures

↓

Discontinuation phase (T0 – Tx)

1. Baseline measurements (T0): thyroid function and secondary outcomes
2. Consultation by GP
3. Dose-lowering of levothyroxine
4. Control measurement of thyroid function after 6 weeks

5. Repetition of steps 2, 3, and 4 until complete discontinuation of levothyroxine:
    - provided that TSH < 10 mIU/L and FT4 within the reference range

↓

Follow-up phase (Tx-T52)

- Final visit 1 year after the start of the discontinuation phase (T52): thyroid function and secondary outcome measures

For peer review only - http://bmjopen.bmj.com/site/about/guidelines.xhtml

Appendix 1

Subject information for participation in medical research

Effects of tapering levothyroxine treatment in elderly subjects: a self-controlled study.
The RELEASE study

Introduction

Dear Sir/Madam,

With this letter, we would like to ask you to take part in a medical study. Participation is voluntary. If you would like to participate, written consent is required. You have received this letter because your general practitioner (GP) has selected you based on your current treatment with artificial thyroid hormone (levothyroxine).

You can take the decision whether you wish to take part in the RELEASE study based on this information sheet which will fully explain this medical study.

- Please, read this information carefully and discuss it with your relatives, friends or family.
- Ask the coordinating physician-investigator or your GP for an explanation if you have any questions.
- You can also ask the independent expert mentioned at the end of this letter for additional information.
- Further information about taking part in a medical research study can be found in the attached brochure 'Medical-scientific research'. It can be found on the government website: https://www.rijksoverheid.nl/documenten/brochures/2014/09/01/medisch-wetenschappelijk-onderzoek-algemene-informatie-voor-de-proefpersoon. You can also request a copy of this brochure from the researcher.

1. General information

This is an investigator-initiated multicentre study. This study was set up at the Department of Public Health & Primary Care Medicine at LUMC by Ms R.K.E. Poortvliet, principal investigator and general practitioner, and Ms A.J.J. Ravensberg, physician-investigator (see appendix A). The study will be conducted by general practitioners, specialists in geriatrics and medical specialists in various general practices, nursing homes and hospitals. It is expected that 360 subjects will participate. The medical ethics review committee Leiden-Den Haag-Delft (METC LDD) has approved this study. General information on research assessment can be found in the brochure 'Medical-scientific research'. The research is funded by a grant from ZonMW.

2. What is the purpose of the study?

The purpose of this study is to find out whether people aged 60 years and older who are taking the drug levothyroxine (thyroid hormone), are treated optimally and whether some of them can reduce or even stop taking levothyroxine.

3. What is the background of the study?

In the Netherlands, many people have been prescribed drugs containing artificial thyroid hormone (levothyroxine). This treatment is started in people with a marked thyroid hormone deficiency, but it

For peer review only - http://bmjopen.bmj.com/site/about/guidelines.xhtml

can also be started in case of a mildly reduced thyroid function. In approximately 15% of all people taking levothyroxine, it is unknown why this drug should be taken.

Levothyroxine treatment is usually continued for life, and is not always monitored regularly. However, there are several reasons why levothyroxine treatment should be monitored regularly and why its usefulness should be reassessed. With age, the body may need less levothyroxine. In addition, some of the reasons to prescribe levothyroxine in the past are now outdated according to current guidelines. For instance, new studies show that levothyroxine treatment later in life does not lead to better health for many individuals with a mildly reduced thyroid function. Moreover, laboratory tests of thyroid function have improved, making it easier today to determine whether levothyroxine treatment is necessary.

Therefore, it is not unexpected that research predicts that a proportion of levothyroxine users aged 60 and above may be treated unnecessarily and thus may not benefit from levothyroxine for their health.

What is the research question?

The RELEASE study investigates how many people aged 60 and older using long-term levothyroxine treatment can gradually discontinue the use of levothyroxine, while maintaining a normal value of thyroid hormone (free T4) and a value of the thyroid stimulating hormone (TSH) of less than 10 mU/L over a period of 1 year. In addition, effects on quality of life and general health will be studied. We will also determine which factors can predict whether levothyroxine treatment can be gradually discontinued while thyroid values remain normal.

Are you eligible to take part?

A total of 360 people aged 60 years and over who have been taking levothyroxine at a stable dose for at least 1 year can participate in this study.

When can you NOT participate in this study?

- If the thyroid gland has been removed.
- If the thyroid gland or neck has been irradiated.
- After treatment with radioactive iodine.
- If there is a thyroid disorder due to use of amiodarone or lithium.
- If you are taking the thyroid hormone liothyronine.
- If you use thiamazole, carbimazole or propylthiouracil (medication that inhibits the thyroid gland).
- If the thyroid disorder is caused by a brain disorder.
- If your last TSH value is equal to or greater than 10 mU/L.
- If your current dose of levothyroxine is higher than 150 micrograms per day
- If you are diagnosed with severe heart failure, dementia or terminal illness.
- If there is incapacity of will.

For peer review only - http://bmjopen.bmj.com/site/about/guidelines.xhtml

4. What happens during the study?

How long will the study take?

Participation in this study takes about 15 months in total.

How will levothyroxine treatment be discontinued?

Your GP will gradually discontinue treatment with levothyroxine in steps, upon agreement with you. This is done partly on the basis of your thyroid function as determined from a blood sample.

Study and measurements

The study consists of a preparation phase, a control phase, a discontinuation phase, a final follow-up, and the collection of medical data.

Preparation

- If you agree to take part in the study, you will be requested to sign a consent form. The signed consent form must be sent to the LUMC research centre.
- You will then be contacted by phone by the physician-investigator or research team member to discuss the study and the completed consent form. If you decide you to participate in the study after this telephone conversation, you will continue with the control phase.

Control phase

Week 1

- You will receive a number of questionnaires at home with general questions about your health, your medication use and your well-being. Please fill these in and send them back. This will take about an hour.
- You will be requested to visit your own GP laboratory to draw blood to check your thyroid function. Preferably, you should visit the laboratory in the morning before you have had breakfast and before you have taken your medication. If necessary, the laboratory can visit you at home.
- You will be requested to discuss the results from the thyroid check with your GP and to continue your usual dose of levothyroxine for 12 weeks.
- However, if your thyroid function is abnormal (free T4 is too low; TSH is too high [≥ 10 mU/L]), you cannot participate in the study. Your GP will then determine which following steps are necessary.

Discontinuation of levothyroxine

Step 1 (week 13)

- You will be requested to complete some questionnaires (this takes about 30 minutes) and to have another blood test to determine thyroid function.
- Your GP will discuss with you whether you are eligible to start discontinuation of levothyroxine. If so, you will receive a prescription for a lower dose of levothyroxine. Collect your new levothyroxine tablets from the pharmacy (or have them delivered) and start taking them.

Step 2

For peer review only - http://bmjopen.bmj.com/site/about/guidelines.xhtml

- Six weeks after the first dose-lowering step, you are requested to fill in questionnaires again (this takes about 20 minutes) and have another blood test to determine thyroid function.
- Based on this check and how you are doing, your GP will discuss with you whether levothyroxine treatment can be further tapered. If so, you will receive a new prescription and continue with lower dose of levothyroxine.

Other steps

- As long as levothyroxine treatment can be further tapered, the dose will be reduced every 6 weeks until it can finally be stopped.
- Monitoring of the thyroid function (in a blood sample) takes place after each dose-lowering step and after stopping. You will also receive questionnaires after stopping levothyroxine (completion takes about 20 minutes).
- How many dose-lowering steps you can complete in total, depends partly on how high your usual dose of levothyroxine was at the start of discontinuation. There is a maximum of 6 dose-lowering steps. The discontinuation phase is estimated to last 2 to 6 months for most participants. In extreme cases, it may take around 10 months.

Final follow-up

- One year after starting dose-lowering of levothyroxine, you will receive the last set of questionnaires to fill in (this takes about 30 minutes). Also you are requested to have a final blood test to determine your thyroid function. Your GP will discuss the results with you.

You will be asked for permission to request medical data from your GP at the start of the study for up to 2 years afterwards. This concerns data from your medical file about your medication use, your medical history, any serious illnesses and/or hospital admissions that occurred during the study period, and any side effects during the discontinuation phase.

5. What agreements do we make with you?

In order for the study to run smoothly, and for your own safety, we want to make the following agreements with you:

- You have blood tests taken to determine your thyroid function several times at your GP's laboratory (at least 4 times). This should preferably be done in the morning before you have had breakfast and before you have taken your levothyroxine. If necessary, a laboratory staff member can visit your home.
- You discuss with your GP, the results of the blood test and the possible tapering of your levothyroxine treatment. This can be done by telephone or during a visit to the general practice.
- You collect a new prescription for a lower dose of levothyroxine with each tapering off step and to start the new dose of levothyroxine as soon as possible.
- You complete 5 or 6 sets of questionnaires at home, and to return these to the research centre before the results of the latest blood sample for thyroid function are known. Completion of the questionnaires takes about 60 minutes for the first set, and 20-30 minutes for each following set.

Additional points of interest:

- This study does not impose restrictions on any of your activities.

For peer review only

- We collect data from your medical records from your GP for the purpose of the study (see bullet nr 4).
- We will inform your GP of your participation in this study.

It is important that you contact the research centre during the study:

- If you are hospitalised.
- If you suddenly develop health problems.
- If you no longer wish to participate in the study.
- If your contact details change.

6. What side effects, adverse effects or discomforts could you experience?

During the discontinuation phase, it is possible that the level of thyroid hormone may become too low (freeT4) or that the level of thyroid stimulation hormone (TSH) may become too high. This may cause symptoms of an inactive thyroid gland such as fatigue, intolerance of cold, muscle ache, constipation and/or a mood change. In this case, your GP will restart levothyroxine or increase the dose.

If you or your GP identify possible side effects, your GP will assess whether you can continue to lower the dose of levothyroxine or not.

7. What are the pros and cons if you take part in the study?

It is important that you carefully weigh up the possible advantages and disadvantages before you decide if you want to participate in this study.

Advantages of participating in the study

- It cannot be guaranteed that you will personally benefit from participating in this study.
- A possible benefit of participation may be that you will eventually no longer need to take levothyroxine or can continue to take a lower dose.
- Your thyroid function will be monitored regularly and your levothyroxine treatment will be reassessed by your GP.
- In the future, the RELEASE study may contribute to improved treatment of thyroid disorders in older adults.

Disadvantages of participating in the study

- It is not expected that you will experience any adverse effects from the blood collection. The total amount of blood collected at each measurement time of the study is a maximum of 10 ml (1 tube of 10 ml).
- You may possibly experience side effects from phasing out levothyroxine: see section 6.

8. What happens if you do not wish to participate or if you wish to stop taking part in the study?

It is up to you to decide whether you wish to participate in the study. Participation is voluntary. If you do not wish to take part, you will be treated with levothyroxine as usual by your GP. If you decide <u>not</u> to participate, no more steps need to be taken. Optionally, you can voluntarily and anonymously return our reply card with the provided envelope. On the reply card, we will ask you about the reason(s) not to take part in the study to improve future scientific research.

If you <u>do</u> take part, you may always change your mind and choose to stop participation, even during the study. You will then be treated as usual with levothyroxine by your GP. You do not have to give a

For peer review only - http://bmjopen.bmj.com/site/about/guidelines.xhtml

reason for stopping. However, you must report stopping to the researcher immediately. The data collected up to that point (including the blood test results) will be used for the study.

The investigator will let you know if there is any new information about the study that is important to you. The investigator will then ask you if you want to continue to take part.

9. When does the study end?

In these situations, the study will stop for you:

- if all the visits described in point 4 are finished,
- if you choose to stop,
- if the investigator or GP considers it better for you to stop,
- if the principal investigator, the government or the reviewing medical ethics committee decides to stop the study.

The entire study ends when all participants have finished and all data have been collected. The GP will discuss with you at the last study visit how to proceed with your medical care (15 months after the start of the study).

After processing all the data, the researcher will inform you and your GP about the main outcomes of the study. This will take place a few months after the last participant has discontinued levothyroxine for 2 years.

10. What will be done with your data?

For this study, your personal data will be used and stored. This includes data such as your name, address, date of birth and data about your health. Blood samples to determine thyroid function are required for this study. The results of these tests are used and stored by the researcher. No blood samples are kept by the researcher. The collection, use, and storage of your data is necessary to answer the research questions of this study and to publish the results. We ask for your consent to use your data.

How do we protect your privacy?

To protect your privacy, your data will be coded. Your name and other data that can directly identify you, are removed. Only the key to the code allows data to be traced back to you. The key to the code remains securely stored at the local research facility. The data sent to the researcher contain only the code, but not your name or any other data that could identify you. In reports and publications about this study, data cannot be traced back to you.

Who can see your data?

Some people can access all your data at the research site, also data without a code. This is necessary to check whether the study has been carried out properly and reliably. Persons who can access this data for control purposes are: the committee monitoring the safety of the study, a monitor of the study, a controller working for the researcher, national supervisory authorities, e.g. the Inspector for Youth and Healthcare. They will keep your data confidential. We will ask you to give permission for this inspection.

For how long do we store your data?

For peer review only - http://bmjopen.bmj.com/site/about/guidelines.xhtml

Your data must be kept at the research site for 15 years.

Can we use your data for other research?

After this study ends, your data may also be relevant to other scientific questions in the field of thyroid disease and levothyroxine treatment. For this purpose, we would like to keep your data for 20 years. You can indicate on the consent form whether or not you agree to this. If you do not agree to this, it is still possible to take part in the RELEASE study. In addition, we ask for your permission to approach you again in the future (after the study has ended) for possible participation in follow-up research.

Can you take back your consent for the use of your data?

You can withdraw your consent to use your personal data at any time. This applies to this study and also to storage and use for possible future research. But please note: if you take back your consent, and the investigators have already collected data for research, they are still allowed to use this information, including the results of the blood tests.

Do you want to know more about your privacy?

For general information on your rights when processing your personal data, please consult the website of the Personal Data Authority or visit https://www.lumc.nl/12367, where you will find more information on LUMC's Privacy Statement. If you have any questions about your rights, please contact the person responsible for processing your personal data. For this study, this is the researcher at the Department of Public Health and Primary Care Medicine at LUMC. See Appendix A for contact details.

If you have any questions or complaints about the processing of your personal data, we advise you to contact the research site first. You can also contact the institution's Data Protection Officer (see Appendix A) or the Personal Data Authority.

Where can you find more information about the study?

Information about this study is also included in a register of medical scientific studies namely www.trialregister.nl. This does not include data traceable to you. After the study, the website may show a summary of the results of this study. You will find this study under 'RELEASE study'.

11. Are you insured during the study?

Insurance has been taken out for everyone taking part in this study. The insurance pays for damage caused by the study. Not all damage is covered. You can find more information about this insurance and any exceptions in Appendix B. It also says who you can report damage to.

12. We will inform your general practitioner

We will always send your GP a message to inform them that you are participating in the study. This is for your own safety. If you do not agree, you cannot take part in this study. Your GP will be informed as soon as you have given consent to participate. The GP has an important role in lowering the dose of levothyroxine and signaling any side effects.

13. Will you receive compensation if you participate in the study?

The additional checks of thyroid function in blood will be reimbursed. You will receive a reimbursement of € 18.50 per blood sample. These checks and contacts with the GP will not affect

For peer review only - http://bmjopen.bmj.com/site/about/guidelines.xhtml

your deductible excess. Transport costs for visits to the GP, pharmacy and/or GP laboratory will not be reimbursed.

14. Do you have any questions?

If you have any questions, please contact your own GP or the coordinating research physician, Ms A.J.J. Ravensberg (LUMC, during office hours, telephone +31 71 526 86 40). You can also send an email to release@lumc.nl. Would you like to get advice from someone who is independent from the study? Then contact the independent expert Ms Dr P.G. van Peet, who can be reached at p.g.van_Peet@lumc.nl or by phone at +31 71 526 844. She is a GP and is aware of all the details of the study, but is not part of this study.

Do you have a complaint? Discuss it with the investigator or the doctor who is treating you. If you prefer not to do so, please visit please contact the LUMC patient service office. Appendix A tells you where to find this.

15. How do you give consent for the study?

You can first think carefully about this study for a period of 4 weeks. Then you tell the investigator if you understand the information and if you want to take part or not. If you want to take part, fill in the consent form that you can find with this information sheet. Your written consent indicates that you have understood the information and agree to participation in the study. You and the investigator will both get a signed version of this consent form.

Thank you for your attention.

16. Appendices to this information

A. Contact details

B. Insurance information

C. Consent forms

Brochure 'RELEASE - Thyroid hormone treatment in people over sixty, a closer look’

For peer review only

A. Contact details RELEASE study

Principal investigator

Ms Dr R.K.E. Poortvliet

Senior general practitioner researcher LUMC

E-mail: r.k.e.poortvliet@lumc.nl

Telephone (during office hours): +31 71 526 8640

Coordinating researcher and first point of contact

Ms A.J.J. Ravensberg

Research physician

E-mail: release@lumc.nl

Telephone (during office hours): +31 71 526 8640

Independent physician

Mrs Dr P.G. van Peet

GP

E-mail: p.g.van_peet@lumc.nl

Telephone (during office hours): +31 71 526 84 44

Complaints

If you do not wish to contact the research team directly, you can always contact the LUMC patient service office.

E-mail: patientenservicebureau@lumc.nl

Telephone (Mon to Fri from 08.30 - 16.30): +31 71 526 2989

Data protection officer of the institution

E-mail: infoavg@lumc.nl

For more information on your rights:

https://www.lumc.nl/12367

For peer review only - http://bmjopen.bmj.com/site/about/guidelines.xhtml

B. INFORMATION TEST SUBJECT INSURANCE

For scientific research with risks in and/or by LUMC

'Effects of tapering treatment with levothyroxine in elderly subjects: a self-controlled study - The RELEASE study'

Protocol number: NL69753.058.19

The LUMC has taken out insurance for everyone who takes part in the study. The insurance pays for any damage you might suffer as a result of participation in this study. This concerns damage due to death or injury, which reveals itself during participation in this research and damage that reveals itself within four years after participation in this research.

The amount covered by the insurance is:

1) €650,000 for damages per subject,

2) €5,000,000 for the damage of all subjects together participating in this study

3) €7,500,000.00 for the total damage manifested per insurance year to test subjects in all investigations the client commissions per insurance year.

Excluded from coverage by this insurance is:

- damage resulting from failure to improve or deterioration of the subject's health problems, if participation in the research is in the context of treatment of these health problems;
- damage due to impairment of the subject's health which is likely to have manifested itself even if the subject had not participated in this investigation.
- damage due to impairment of the subject's health if such damage results from participation in a scientific study consisting of a comparison of two or more usual treatments or procedures;
- damage manifesting itself in offspring as a result of an adverse effect of the research on the subject and/or the offspring;
- damage of which it is certain or virtually certain by the nature of the research that it would occur in the subject;
- damage resulting from the subject's failure to comply or not fully comply with directions and instructions, insofar as the subject is capable of doing so;
- damage resulting from the occurrence of a risk for which you were warned in the written information, unless the risk occurs to a greater extent than was foreseen or the risk was extremely unlikely.

The name of the insurance company where the study is insured is:

Onderlinge Waarborgmaatschappij Centramed B.A.
Appelgaarde 4
2272 TK Voorburg

If one has suffered or suspects damage, one should contact the investigator or the treating physician as soon as possible.

For peer review only - http://bmjopen.bmj.com/site/about/guidelines.xhtml

C. Subject consent form 'Effects of tapering treatment with levothyroxine in elderly subjects: a self-controlled study - The RELEASE study'

- I have read the information letter. I was also able to ask questions. My questions have been answered well enough. I had enough time to decide if I wanted to take part.
- I know that taking part is voluntary. I also know that at any time I can decide not to take part in the study, or to stop taking part. I do not have to explain why.
- I give the investigator consent to inform my treating physician (GP, geriatric specialist or hospital doctor) that I am taking part in this study.
- I give consent to request medical information from my treating physician for the purpose of the study.
- I give consent to collect and use my data and the results of my blood tests for answering the research question in this study.
- I know that some people will be able to see all my data to review the study. These people are listed in this information letter. I give consent to let them see my data for this review.
- I give consent to keep my research data for 15 years after the end of this study.
- I do □ / do not □
  give consent to keep and use my personal data longer for future research in the field of thyroid disorders and levothyroxine treatment, namely for the duration of 20 years. *
- I do □ / do not □
  give consent to the investigator to contact me for a follow-up study after this study. *
- I want to take part in this study.

* Check the relevant box

Data subject

Name Mr/Mrs .... Date of birth ....

Address .... Postal code.....................................

City .... Phone number ....

Signature .... Date ....

____

To be completed by the research officer of RELEASE study

I declare that I have fully informed this subject about the study mentioned.

If any information becomes known during the study that could influence the subject's consent, I will let this subject know in good time.

Investigator name (or their representative):

Signature: Date: __ / __ / __

Additional information was given by (if applicable):

For peer review only - http://bmjopen.bmj.com/site/about/guidelines.xhtml

Name:

Job title:

Signature: Date: __ / __ / __

The study subject will receive a complete information sheet, together with a signed version of the consent form.

For peer review only

For peer review only - http://bmjopen.bmj.com/site/about/guidelines.xhtml

Appendix 2 – Outcomes

Instruments used to determine baseline characteristics and secondary outcome parameters

Integrated Systematic Care for Older Persons (ISCOPE) Questionnaire

The ISCOPE questionnaire indicates the degree of complex problems in older participants by measuring problems in the functional, somatic, psychological and social domain as experienced by older persons (1, 2). This questionnaire consists of 26 closed questions. The score ranges from 0 (no problems in those domains) to 4 (problems on 4 domains).

Revised Patients' Attitudes Towards Deprescribing (rPATD) questionnaire

The revised Patients 'Attitudes Towards Deprescribing (rPATD) questionnaire was developed to capture older adults' and caregivers' beliefs and attitudes towards deprescribing (3). The rPATD was developed and validated for older adults ≥65 years of age taking at least one regular medication and caregivers of older adults. The revised PATD questionnaire had acceptable validity and reliability.

Treatment satisfaction (TSQM)

The Treatment Satisfaction Questionnaire for Medication version II (TSQM) was developed as a measure of treatment satisfaction for medication without disease-specific focus (4). This allows the questionnaire to be well fitted to a range of participant conditions and characteristics. Furthermore it allows for sound comparing of treatment satisfaction with other medication or medication discontinuation studies. The TSQM has been validated in multiple languages (including Dutch) and is reported to have sound psychometric properties. It consists of 11 questions, all using 7-point Likert scales.

Goal Attainment Scale (GAS)

The GAS questionnaire will be used to ask participants what dose reduction of levothyroxine treatment they would define as a substantial dose reduction (at pre-baseline), and whether they achieved this (at final follow-up).

Thyroid-specific quality of life (ThyPro39)

The ThyPRO39 is a questionnaire covering a comprehensive range of thyroid-related quality of life issues and considerations spanning somatic, psychological and social domains. It consists of 84 questions, all 5-point Likert scale, ranging 13 thyroid-specific quality of life domains. In a systematic review the ThyPRO was nominated as the recommended method for assessing health-related quality of life in benign thyroid diseases (5). Measuring ThyPRO39 at baseline, 6 weeks after the start of the

For peer review only

discontinuation phase and at the end of the discontinuation phase, will give insight to symptoms that may have triggered stopping the discontinuation phase.

Quality of life (EQ5D)

The EQ5D questionnaire is a standardized instrument for use as a measure of health outcome (6). The Visual Analogue Scale is a horizontal line ranging from 0 (worst imaginable health) to 100 (perfect health) on which the patients mark the point that they feel represents their perception of their current health state.

Decision Regret Scale (DRS)

The Decision Regret Scale is a validated indicator of health care decision regret at a given point in time (7). It will be used at the final follow-up to evaluate regret after the decision to withdraw levothyroxine treatment. The questionnaire consists of 5 items. The participant indicates for each statement the extent to which they agree or disagree. Scoring consists of reversing the scores of the 2 negatively phrased items (2 and 4), then taking the mean of the 5 items. These means can be converted to a score ranging from 0 to 100 by subtracting 1 and multiplying by 25.

References

1. Blom J, den Elzen W, van Houwelingen AH, Heijmans M, Stijnen T, Van den Hout W, et al. Effectiveness and cost-effectiveness of a proactive, goal-oriented, integrated care model in general practice for older people. A cluster randomised controlled trial: Integrated Systematic Care for older People--the ISCOPE study. Age Ageing. 2016;45(1):30-41.
2. van Houwelingen AH, den Elzen WP, le Cessie S, Blom JW, Gussekloo J. Consequences of interaction of functional, somatic, mental and social problems in community-dwelling older people. PLoS One. 2015;10(4):e0121013.
3. Reeve E, Low LF, Shakib S, Hilmer SN. Development and Validation of the Revised Patients' Attitudes Towards Deprescribing (rPATD) Questionnaire: Versions for Older Adults and Caregivers. Drugs Aging. 2016;33(12):913-28.
4. Atkinson MJ, Kumar R, Cappelleri JC, Hass SL. Hierarchical construct validity of the treatment satisfaction questionnaire for medication (TSQM version II) among outpatient pharmacy consumers. Value Health. 2005;8 Suppl 1:S9-s24.
5. Wong CK, Lang BH, Lam CL. A systematic review of quality of thyroid-specific health-related quality-of-life instruments recommends ThyPRO for patients with benign thyroid diseases. J Clin Epidemiol. 2016;78:63-72.

For peer review only

6. EuroQol. EuroQol--a new facility for the measurement of health-related quality of life. Health Policy. 1990;16(3):199-208.

7. Brehaut JC, O'Connor AM, Wood TJ, Hack TF, Siminoff L, Gordon E, et al. Validation of a decision regret scale. Med Decis Making. 2003;23(4):281-92.

For peer review only

Appendix 3. Trial information

Trial registration data

**Data category**	**Information**
Primary registry and trial identifying number	Netherlands Trial Register NL7978
Date of registration in primary registry	22 August, 2019
Secondary identifying numbers	ZonMW projectnumber 839110026
Source(s) of monetary or material support	ZonMW HGOG
Primary sponsor	Leiden University Medical Center
Secondary sponsor(s)	Not applicable
Contact for public queries	R.K.E. Poortvliet, MD, PhD – Leiden University Medical Center – release@lumc.nl
Contact for scientific queries	R.K.E. Poortvliet, MD, PhD – Leiden University Medical Center – r.k.e.poortvliet@lumc.nl
Public title	REduction of LEvothyroxine in Adults; a SElf-controlled study (RELEASE)
Scientific title	Effect of discontinuation of levothyroxine treatment in older adults; a self-controlled study
Countries of recruitment	The Netherlands
Health condition(s) or problem(s) studied	Hypothyroidism
Intervention(s)	Stepwise reduction of levothyroxine treatment
Key inclusion and exclusion criteria	Inclusion criteria:<br>1) Aged 60 years or over (= 60 years)<br>2) Using any levothyroxine mono-therapy medicament (ATC: H03AA01) continuously for a minimum of 1 year with stable dose of levothyroxine.<br>Exclusion criteria:<br>1) Last measurement of TSH =10 mU/L during levothyroxine treatment<br>2) Current reason for levothyroxine treatment: patients with history of thyroidectomy; radioactive iodine treatment or neck irradiation; congenital hypothyroidism; secondary hypothyroidism, or concurrent amiodarone or lithium use<br>3) Dose of treatment; for safety issues, patients using > 150 mcg levothyroxine per day will not be eligible<br>4) Diagnosis of heart failure NYHA grade IV<br>5) Participation in ongoing trials of therapeutic interventions<br>6) Life-expectancy of less than 6 months<br>7) Diagnosis of dementia<br>8) Incapacitated adults<br>9) Persons that plan to move out of the region

For peer review

	in which the study is being conducted in the next months.
Study type	Observational
Date of first enrolment	13-02-2020*
Target sample size	385*
Recruitment status	Active, not recruiting*
Primary outcome(s)	The proportion of participants that withdraw their thyroid medication successfully defined as normal fT4 levels and TSH levels <10 mU/L at 52 weeks after the start of the discontinuation.
Key secondary outcome(s)	1. The proportion of participants that achieve a substantial dose reduction of levothyroxine at 52 weeks a) defined as ≥50% and b) defined by the participants themselves; 2. The effect of discontinuation of levothyroxine treatment at 52 weeks on a) thyroid-related quality of life and b) general health; 3. How participants reflect on their decision to discontinue levothyroxine treatment.

* These are updated data. The Netherlands Trial Register is no longer available. Trial data registered in the NTR can be consulted on the International Clinical Trial Registry Platform (ICTRP) Search Portal, but it is no longer possible to adjust these data. This study will be re-registered at ClinicalTrials.gov to keep an up to date trial registration.

Trial sponsor

Leiden University Medical Center

Sponsor's reference: NL69753.058.19

Contact name: Prof. dr. J. Gussekloo

Address: Leiden University Medical Center, Department of Public Health and Primary Care

Postzone V-0-P; PO BOX 9600; 2300 RC Leiden, the Netherlands

Telephone: +31 715268444

Email: j.gussekloo@lumc.nl

Roles and responsibilities – committees

Principal investigators: J. Gussekloo, R.K.E. Poortvliet, R.S. Du Puy

- Responsible for the design and conduct of the study

Coordinating investigator /project leader: R.K.E. Poortvliet and Research physician: A.J. Ravensberg

- Preparation of protocol and revisions
- Preparation of investigators brochure (IB) and case report forms (CRFs)
- Organising steering committee meetings
- Publication of study reports

For peer review only

Steering committee (SC): J. Gussekloo, R.K.E. Poortvliet, R.S. Du Puy; A.J. Ravensberg; O.M. Dekkers; S.P. Mooijaart

- Agreement of final protocol
- Reviewing progress of study and if necessary agreeing changes to the protocol and/or investigators brochure to facilitate the smooth running of the study.

Trial management committee (TMC)

- Members: coordinating investigator R.K.E. Poortvliet and research physician A.J. Ravensberg
- Study planning
- Organization of SC meetings
- Establish regular reports for the Institutional Ethics Committee and the Data Safety Monitoring Board (DSMB)
- SAE [serious adverse event] and SUSAR [serious unexpected suspected adverse events] reporting to the Dutch Central Committee on Research Involving Human Subjects (CCMO) and to the DSMB
- Responsible for trial master file
- Budget administration and contractual issues with individual centers (general practices)
- Advice for lead investigators
- Preparing and follow-up of trial audits
- Assistance with international review, board/independent ethics committee applications
- Data verification
- Organisation of data collection

Data management: A.J. Ravensberg; B.M. Schakenbos; H. de Jong

- Maintenance of trial IT system and data entry
- Data verification

For peer review only

STANDARD PROTOCOL ITEMS: RECOMMENDATIONS FOR INTERVENTIONAL TRIALS

SPIRIT 2013 Checklist: Recommended items to address in a clinical trial protocol and related documents*

Section/item	Item No	Description	Addressed on page number
**Administrative information**			
Title	1	Descriptive title identifying the study design, population, interventions, and, if applicable, trial acronym	Page 1
Trial registration	2a	Trial identifier and registry name. If not yet registered, name of intended registry	Page 2
	2b	All items from the World Health Organization Trial Registration Data Set	Appendix 1 – Trial information
Protocol version	3	Date and version identifier	Page 2
Funding	4	Sources and types of financial, material, and other support	Page 14
Roles and responsibilities	5a	Names, affiliations, and roles of protocol contributors	Page 1 and 14
	5b	Name and contact information for the trial sponsor	Appendix 1 Trial information
	5c	Role of study sponsor and funders, if any, in study design; collection, management, analysis, and interpretation of data; writing of the report; and the decision to submit the report for publication, including whether they will have ultimate authority over any of these activities	Page 14

For peer review only

For peer review only - http://bmjopen.bmj.com/site/about/guidelines.xhtml

	5d	Composition, roles, and responsibilities of the coordinating centre, steering committee, endpoint adjudication committee, data management team, and other individuals or groups overseeing the trial, if applicable (see Item 21a for data monitoring committee)	Appendix 1 Trial information
**Introduction**			
Background and rationale	6a	Description of research question and justification for undertaking the trial, including summary of relevant studies (published and unpublished) examining benefits and harms for each intervention	Page 4 and 5
	6b	Explanation for choice of comparators	Page 5 and 6
Objectives	7	Specific objectives or hypotheses	Page 5
Trial design	8	Description of trial design including type of trial (eg, parallel group, crossover, factorial, single group), allocation ratio, and framework (eg, superiority, equivalence, noninferiority, exploratory)	Page 5
**Methods: Participants, interventions, and outcomes**			
Study setting	9	Description of study settings (eg, community clinic, academic hospital) and list of countries where data will be collected. Reference to where list of study sites can be obtained	Page 6
Eligibility criteria	10	Inclusion and exclusion criteria for participants. If applicable, eligibility criteria for study centres and individuals who will perform the interventions (eg, surgeons, psychotherapists)	Page 6-7
Interventions	11a	Interventions for each group with sufficient detail to allow replication, including how and when they will be administered	Page 7-8
	11b	Criteria for discontinuing or modifying allocated interventions for a given trial participant (eg, drug dose change in response to harms, participant request, or improving/worsening disease)	Page 8
	11c	Strategies to improve adherence to intervention protocols, and any procedures for monitoring adherence (eg, drug tablet return, laboratory tests)	Page 8
	11d	Relevant concomitant care and interventions that are permitted or prohibited during the trial	Not applicable.

Outcomes	12	Primary, secondary, and other outcomes, including the specific measurement variable (eg, systolic blood pressure), analysis metric (eg, change from baseline, final value, time to event), method of aggregation (eg, median, proportion), and time point for each outcome. Explanation of the clinical relevance of chosen efficacy and harm outcomes is strongly recommended	Page 8-9, 11, Appendix 3 - Outcomes
Participant timeline	13	Time schedule of enrolment, interventions (including any run-ins and washouts), assessments, and visits for participants. A schematic diagram is highly recommended (see Figure)	Figure 1
Sample size	14	Estimated number of participants needed to achieve study objectives and how it was determined, including clinical and statistical assumptions supporting any sample size calculations	Page 11
Recruitment	15	Strategies for achieving adequate participant enrolment to reach target sample size	Page 6

Methods: Assignment of interventions (for controlled trials)

Allocation:

Sequence generation	16a	Method of generating the allocation sequence (eg, computer-generated random numbers), and list of any factors for stratification. To reduce predictability of a random sequence, details of any planned restriction (eg, blocking) should be provided in a separate document that is unavailable to those who enrol participants or assign interventions	Not applicable
Allocation concealment mechanism	16b	Mechanism of implementing the allocation sequence (eg, central telephone; sequentially numbered, opaque, sealed envelopes), describing any steps to conceal the sequence until interventions are assigned	Not applicable
Implementation	16c	Who will generate the allocation sequence, who will enrol participants, and who will assign participants to interventions	Page 6-7
Blinding (masking)	17a	Who will be blinded after assignment to interventions (eg, trial participants, care providers, outcome assessors, data analysts), and how	Not applicable
	17b	If blinded, circumstances under which unblinding is permissible, and procedure for revealing a participant's allocated intervention during the trial	Not applicable

Methods: Data collection, management, and analysis

Data collection methods	18a	Plans for assessment and collection of outcome, baseline, and other trial data, including any related processes to promote data quality (eg, duplicate measurements, training of assessors) and a description of study instruments (eg, questionnaires, laboratory tests) along with their reliability and validity, if known. Reference to where data collection forms can be found, if not in the protocol	Page 8-9, Appendix 3 Outcomes
	18b	Plans to promote participant retention and complete follow-up, including list of any outcome data to be collected for participants who discontinue or deviate from intervention protocols	Page 8
Data management	19	Plans for data entry, coding, security, and storage, including any related processes to promote data quality (eg, double data entry; range checks for data values). Reference to where details of data management procedures can be found, if not in the protocol	Page 11
Statistical methods	20a	Statistical methods for analysing primary and secondary outcomes. Reference to where other details of the statistical analysis plan can be found, if not in the protocol	Page 11-12
	20b	Methods for any additional analyses (eg, subgroup and adjusted analyses)	Page 11-12
	20c	Definition of analysis population relating to protocol non-adherence (eg, as randomised analysis), and any statistical methods to handle missing data (eg, multiple imputation)	Page 11-12
**Methods: Monitoring**			
Data monitoring	21a	Composition of data monitoring committee (DMC); summary of its role and reporting structure; statement of whether it is independent from the sponsor and competing interests; and reference to where further details about its charter can be found, if not in the protocol. Alternatively, an explanation of why a DMC is not needed	Page 12
	21b	Description of any interim analyses and stopping guidelines, including who will have access to these interim results and make the final decision to terminate the trial	Page 12
Harms	22	Plans for collecting, assessing, reporting, and managing solicited and spontaneously reported adverse events and other unintended effects of trial interventions or trial conduct	Page 12
Auditing	23	Frequency and procedures for auditing trial conduct, if any, and whether the process will be independent from investigators and the sponsor	Page 12
**Ethics and dissemination**			

For peer review only - http://bmjopen.bmj.com/site/about/guidelines.xhtml

Research ethics approval	24	Plans for seeking research ethics committee/institutional review board (REC/IRB) approval	Page 14
Protocol amendments	25	Plans for communicating important protocol modifications (eg, changes to eligibility criteria, outcomes, analyses) to relevant parties (eg, investigators, REC/IRBs, trial participants, trial registries, journals, regulators)	Page 14
Consent or assent	26a	Who will obtain informed consent or assent from potential trial participants or authorised surrogates, and how (see Item 32)	Page 6
	26b	Additional consent provisions for collection and use of participant data and biological specimens in ancillary studies, if applicable	Not applicable.
Confidentiality	27	How personal information about potential and enrolled participants will be collected, shared, and maintained in order to protect confidentiality before, during, and after the trial	Page 11
Declaration of interests	28	Financial and other competing interests for principal investigators for the overall trial and each study site	Page 14
Access to data	29	Statement of who will have access to the final trial dataset, and disclosure of contractual agreements that limit such access for investigators	Page 11
Ancillary and post-trial care	30	Provisions, if any, for ancillary and post-trial care, and for compensation to those who suffer harm from trial participation	Page 14
Dissemination policy	31a	Plans for investigators and sponsor to communicate trial results to participants, healthcare professionals, the public, and other relevant groups (eg, via publication, reporting in results databases, or other data sharing arrangements), including any publication restrictions	Page 14
	31b	Authorship eligibility guidelines and any intended use of professional writers	Page 14
	31c	Plans, if any, for granting public access to the full protocol, participant-level dataset, and statistical code	Page 14

Appendices

For peer review only - http://bmjopen.bmj.com/site/about/guidelines.xhtml

Informed consent materials	32	Model consent form and other related documentation given to participants and authorised surrogates	Appendix 2 Subject information sheet and consent form
Biological specimens	33	Plans for collection, laboratory evaluation, and storage of biological specimens for genetic or molecular analysis in the current trial and for future use in ancillary studies, if applicable	Not applicable

*It is strongly recommended that this checklist be read in conjunction with the SPIRIT 2013 Explanation & Elaboration for important clarification on the items. Amendments to the protocol should be tracked and dated. The SPIRIT checklist is copyrighted by the SPIRIT Group under the Creative Commons "Attribution-NonCommercial-NoDerivs 3.0 Unported" license.

For peer review only
